# Syndromic Analysis of Sepsis Cohorts Using Large Language Models

**DOI:** 10.1001/jamanetworkopen.2025.39267

**Published:** 2025-10-24

**Authors:** Theodore R. Pak, Sanjat Kanjilal, Caroline S. McKenna, Alexander Hoffner-Heinike, Chanu Rhee, Michael Klompas

**Affiliations:** 1 Department of Population Medicine, Harvard Medical School and Harvard Pilgrim Health Care Institute, Boston, Massachusetts; 2 Division of Infectious Diseases, Department of Medicine, Massachusetts General Hospital, Boston; 3 Division of Infectious Diseases, Department of Medicine, Brigham and Women’s Hospital, Boston, Massachusetts; 4 Department of Medicine, Massachusetts General Hospital, Boston

## Abstract

**Importance:**

Presenting signs and symptoms affect the care of patients with possible sepsis. However, signs and symptoms are not incorporated into most large observational studies because they are difficult to extract from clinical notes at scale.

**Objective:**

To assess the use of large language models (LLMs) to extract presenting signs and symptoms from admission notes and characterize their associations with infectious diagnoses, multidrug-resistant infections, and mortality.

**Design, Setting, and Participants:**

This retrospective cohort study obtained data from 5 Massachusetts hospitals within 1 health care system between June 1, 2015, and August 1, 2022. Participants were hospitalized adult patients with possible infection (determined by blood culture drawn and intravenous antibiotics administered within 24 hours of arrival). An LLM (LLaMA 3 8B; Meta) was used to extract up to 10 presenting signs and symptoms from each patient’s history-and-physical admission notes. LLM-generated labels were validated by blinded review of 303 random admission notes. Data analyses were performed from July 2023 to August 2025.

**Exposures:**

Thirty most common signs and symptoms were retained as exposures, and unsupervised clustering was used to create syndromes, which were compared with infection sources derived from the *International Statistical Classification of Diseases, Tenth Revision, Clinical Modification* discharge codes.

**Main Outcomes and Measures:**

Outcomes included positive cultures for methicillin-resistant *Staphylococcus aureus* (MRSA), positive cultures for multidrug-resistant gram-negative (MDRGN) organisms, and in-hospital mortality. Multivariable logistic regression was used to adjust for demographics, comorbidities, physiologic markers of severity of illness, and time to antibiotics.

**Results:**

Among the 104 248 patients (median [IQR] age, 66 [52-78] years; 54 137 males [51.9%]) included, 23 619 (22.7%) had sepsis without shock, 25 990 (24.9%) had septic shock, and 94 913 (91.0%) had 1 or more admission note within 24 hours. The LLM labeled the notes of 93 674 of 94 913 patients (98.7%). On manual validation, LLM labels had an accuracy of 99.3% (95% CI, 99.2%-99.3%), balanced accuracy of 84.6% (95% CI, 83.5%-85.8%), positive predictive value of 68.4% (95% CI, 66.0%-70.7%), sensitivity of 69.7% (95% CI, 67.3%-72.0%), and specificity of 99.6% (95% CI, 99.6%-99.6%) compared with the physician medical record reviewer. The 30 most common signs and symptoms were clustered into syndromes that correlated with infection sources. Presence of skin and soft tissue symptoms (adjusted odds ratio [AOR], 1.73; 95% CI, 1.49-2.00) and absence of gastrointestinal (AOR, 0.63; 95% CI, 0.54-0.73) or urinary tract symptoms (AOR, 0.34; 95% CI, 0.22-0.50) were associated with MRSA culture positivity; inverse associations were seen for MDRGN organisms. Cardiopulmonary symptoms were associated with increased mortality (AOR, 1.30; 95% CI, 1.17-1.45).

**Conclusions and Relevance:**

This cohort study found that an LLM accurately extracted presenting signs and symptoms from admission notes that clustered into syndromes differentially correlated with infection sources, multidrug-resistant infections, and mortality. Further research is warranted to evaluate the value of large-scale sign-and-symptom data in models of antibiotic choice, effectiveness, and outcomes in patients with possible sepsis.

## Key Points

**Question:**

Can large language models (LLMs) accurately extract presenting signs and symptoms from clinical notes to identify associations between symptoms, multidrug-resistant infections, and in-hospital mortality in large cohorts of patients with possible sepsis?

**Findings:**

In this cohort study of 104 248 patients with possible infection, LLMs extracted signs and symptoms from admission notes with accuracy comparable to that of physicians performing manual medical records review. Hierarchical clustering identified 7 symptom-based syndromes that correlated with infection sources, risk for methicillin-resistant *Staphylococcus aureus* and multidrug-resistant gram-negative organisms, and in-hospital death.

**Meaning:**

Findings of this study suggest that LLMs can enable the efficient, large-scale extraction of signs and symptoms from clinical notes and the differential correlation of syndromes with infection sources, multidrug-resistant infections, and mortality; the value of large-scale sign-and-symptom data in models of antibiotic choice, effectiveness, and outcomes in patients with sepsis warrants further study.

## Teaser

This cohort study compares the performance of a large language model against manual medical record review for extracting presenting signs and symptoms data from hospital admission notes and uses these data to examine their associations with different sepsis syndromes and mortality.

## Introduction

Sepsis contributes to 20% of all deaths globally.^1^ It also plays a major role in broad-spectrum antimicrobial use, in part because treatment guidelines and quality metrics recommend broad-spectrum antibiotics for all patients with possible sepsis within 1 to 3 hours of recognition.^2,3,4^ Empiric treatment often includes coverage for methicillin-resistant *Staphylococcus aureus* (MRSA) and multidrug-resistant gram-negative (MDRGN) bacteria, but only 5% to 14% of patients with positive cultures have evidence of any of these resistant organisms.^5,6,7^ Overly broad or unnecessary antibiotic use is potentially harmful. Up to 20% of hospitalized patients experience antibiotic adverse effects,^8^ and overly broad antibiotic regimens are associated with increased mortality.^5^

Clinicians seeing patients with possible sepsis or septic shock typically have little information on which to base initial treatment decisions, as definitive diagnostic information typically takes hours or days to return.^9^ Consequently, clinicians often rely on patients’ presenting signs and symptoms to decide whether and which antibiotics to use. Notwithstanding the importance of signs and symptoms to inform initial antibiotic choices, most large cohort studies assessing the associations between antibiotic choice, timing, and outcomes have not included presenting signs and symptoms as potentially informative variables.^5,7,10,11,12,13,14,15,16,17,18,19^ This gap is likely because signs and symptoms are usually recorded in unstructured clinical notes and require laborious and subjective medical record reviews to extract. Smaller studies that did use medical record review have found that certain symptoms are associated with lower SEP-1 (Severe Sepsis and Septic Shock Early Management Bundle) compliance^20^ and higher mortality,^21^ but scalable approaches to extract these data have been lacking. *International Statistical Classification of Diseases, Tenth Revision, Clinical Modification* (*ICD-10-CM*) codes capture some symptom data (eg, codes R00-R99), but these codes are inconsistently applied and typically superseded by more specific diagnoses whenever possible. The lack of large-scale symptom data has also complicated efforts to find subphenotypes of sepsis that could guide more individualized care. To date, sepsis subphenotyping studies either describe clusters of vital signs and laboratory data not recognizable to clinicians^22,23,24^ or require the collection of biomarkers and trajectory data not routinely available within the first few hours of presentation.^25,26^

Large language models (LLMs) can adeptly manipulate unstructured text with minimal pretraining and offer a promising new strategy to extract structured data, such as presenting signs and symptoms, from clinical notes at scale. Despite most LLMs being designed for general rather than medical use, they have matched human performance at medical board examination–style questions^27,28^ and complex diagnostic challenges,^29,30^ and they are showing promise as accurate information extractors for clinical text.^31,32,33,34^ Nevertheless, LLMs have well-described limitations, including uncertain reproducibility, uninterpretable behavior, and hidden biases.^35,36,37^ Few studies have investigated LLMs’ ability to extract data from thousands of uncurated clinical notes containing protected health information.

In this study, we aimed to assess the use of LLMs to extract presenting signs and symptoms from admission notes and characterize their associations with infectious diagnoses, multidrug-resistant infections, and mortality in a large cohort of patients with possible infection. We evaluated whether distinct symptom-based clusters were associated with specific pathogens, antibiotic-resistance profiles, and mortality and compared the performance of the LLM-based approach with the performance of the *ICD-10-CM* method using manual medical record reviews as ground truth. Our goal was to develop and validate a scalable approach to capturing symptom data that serves as the groundwork for improved predictive models of pathogen categories, antimicrobial resistance profiles, patient prognoses, and tailored empiric antibiotic prescribing for sepsis.

## Methods

### Study Design, Data Source, and Population

This retrospective cohort study included all adults aged 18 years or older who were admitted via the emergency department (ED) to 5 hospitals in the Mass General Brigham (MGB) health care system from June 1, 2015, to August 1, 2022. All data were collected from an electronic health record data warehouse (Clarity; Epic Systems) and laboratory information systems. All computational analysis involving protected health information used MGB’s Health Insurance Portability and Accountability Act–compliant high-performance computing clusters. The MGB Institutional Review Board deemed this study exempt from review and waived the informed consent requirement because the use of identifiable health information was for research purposes. We followed the Strengthening the Reporting of Observational Studies in Epidemiology (STROBE) reporting guideline.

We included all patients with possible infection, defined as patients who underwent collection of 1 or more blood cultures and administration of intravenous (IV) antibiotics within 24 hours of ED arrival. Exclusion criteria included patients who received comfort measures or died within 6 hours of arrival, were transferred from outside hospitals, were admitted to psychiatry or obstetric services, were missing key laboratory test results or vital signs within 12 hours of ED arrival, or had previously received antibiotics.^17^

### LLM Extraction of Presenting Signs and Symptoms

For each admission, we extracted all history-and-physical admission notes with a time of service within 24 hours of ED arrival. We used regular expressions to detect the start and end of History of Present Illness (HPI) sections (eMethods in Supplement 1), extracted their contents, and incorporated them into an LLM prompt (Box).

Box. **Large Language Model Prompt for Extracting Presenting Signs and Symptoms**

**System:** You are a clinical researcher that reads medical charts and answers questions about them. Use only the information in the text provided to answer the question. If a patient denies something, do not include it in your answer. After you provide an answer, you immediately stop talking.

**User:** Read the following patient history and list the patient’s presenting symptoms. Include only symptoms present now or reported for the days to weeks leading up to admission. Ignore any symptoms from past medical history or prior hospital admissions. Give your answer as a JSON array containing up to ten strings. Each string contains between one and three words.

[History of Present Illness contents inserted here]

**Assistant:** …

The vLLM Python framework for high-throughput inference^38^ was used to process these prompts through the publicly downloadable LLaMA 3 model, 8B version (Meta),^39^ hereafter referred to as the LLM. Model weights were cast to the float16 format from the LLM’s native bfloat16 format, but no further weight modification or fine-tuning was performed. We ran inference on computer servers with Intel Xeon Gold 6148 central processing units running at 2.40 GHz and Nvidia Tesla V100 GPUs (graphics processing units) with 32 GB of memory, with each GPU holding a single instance of the model. The context window was 3044 tokens, and token sampling was performed using temperature 0.1, top-p 0.8, repetition penalty 1.05, and constrained decoding to ensure the output was a valid array of strings in JavaScript Object Notation.^40^ An alternative prompting strategy, where the model was prompted with 5 specific signs and symptoms at a time, was also implemented (eMethods in Supplement 1).

### Labeling Admission Notes Using a Controlled Vocabulary of Signs and Symptoms

We derived a controlled vocabulary of signs and symptoms as follows. First, we created a large corpus of sign-and-symptom text by running a preliminary version of the LLM extraction process on all notes for the cohort. Second, from this corpus, we sorted all words, bigrams, and trigrams by descending frequency; excluded stop words (common English words with less information such as *the* and *in*) using the tm package in R; and filtered terms to the top 75% of the distribution to generate a set of candidate terms. Third, we manually filtered out terms that were not fully conveying a sign and symptom (eg, *failure* and *feeling*). Fourth, to create a controlled vocabulary, we manually merged synonymous terms (eg, *decreased urine output* and *oliguria*) and selected canonical terms, preferring terms with the broadest consistent meaning among grouped terms (eg, *redness* over *erythema*). Fifth, to label each admission note with vocabulary terms, we scanned LLM-generated strings for matching terms using a backoff strategy, that is, longer n-gram matches took precedence over shorter n-grams.

For comparison with a non-LLM method for labeling admission notes with presenting signs and symptoms, we extracted all *ICD-10-CM* codes for clinicians’ daily professional charges from 7 days prior to admission up to and including the date of admission. We then used a mapping of *ICD-10-CM* codes for the 30 most common signs and symptoms in this cohort (eTable 1 in Supplement 1) to label admissions.

### Validation of Sign and Symptom Labels Generated by the LLM vs Physician Medical Record Review

A random sample of 303 admission notes underwent manual review by an infectious disease fellow (T.R.P.), who extracted presenting signs and symptoms using the same instructions as the LLM prompt. Notes were presented to reviewers using a custom web interface that displayed only the text of the admission notes and a drop-down list for selecting up to 10 signs and symptoms from the vocabulary, blinding reviewers to physician-generated and LLM-generated labels and any patient data outside the admission notes. Synonyms were automatically remapped by the website to corresponding canonical terms. To measure interrater reliability, from the first random sample of 303 notes, a second random sample of 100 notes was selected for manual review by an internal medicine resident (A.H.-H.), who was blinded to the LLM-generated and physician-generated labels.

### Statistical Analysis

Labeling performance of the LLM method against that of a physician reviewer and of 2 physician reviewers against each other was assessed by calculating microaveraged accuracy, balanced accuracy, sensitivity, specificity, and Cohen κ, using either Wilson score intervals or 1000 bootstraps to calculate 95% CIs. We also calculated positive predictive values, negative predictive values, and F1 scores for each comparison, using Wilson score intervals or the Takahashi method for F1 scores^41^ to construct 95% CIs. We similarly assessed the labeling performance of the *ICD-10-CM* method. For parity, these comparisons filtered LLM-generated and physician-generated labels to the smaller set of labels available to the *ICD-10-CM* method.

After ranking all 404 sign and symptom labels by descending frequency in the labeled cohort, the 30 most common signs and symptoms were selected for further analysis. Pearson correlation coefficients (*r*) were calculated for the co-occurrence of each pair of signs and symptoms in patient admissions. To cluster symptoms into syndromes, we performed single-linkage hierarchical clustering of the vectors of Pearson *r* by Euclidean distance, followed by tree cutting into *k* = 7 clusters, selecting *k* to correspond to the number of body sites in a previously defined categorization of *ICD-10-CM* codes that identified infection sources.^5,17^ Clusters were named using the consensus judgment of the 5 study physicians (T.R.P., S.K., A.H.-H., C.R., and M.K.). To verify correspondence of syndromes with specific infection sources, we calculated correlations between each sign and symptom and the categorizations of present-on-admission *ICD-10-CM* discharge codes.^5,17^

We examined associations between presenting signs and symptoms and 3 outcomes: in-hospital mortality, isolation of MRSA from at least 1 clinical culture collected within 72 hours of ED arrival, and isolation of an MDRGN organism (including ceftriaxone-resistant gram-negative bacteria, ampicillin-sulbactam–resistant *Acinetobacter*, and AmpC-producing *Enterobacterales*) from at least 1 culture collected within 72 hours of ED arrival (eMethods in Supplement 1). We calculated crude relative risks (RRs) for the association between each sign and symptom and each outcome variable by comparing the proportions of patients with cultures positive for MRSA and MDRGN organisms having each sign or symptom against the proportions of all patients with MRSA and MDRGN organisms, while in-hospital mortality was compared among all patients labeled with possible infection. We then fit multivariable logistic regression models to estimate adjusted odds ratios (AORs) for each outcome variable vs presence of 1 or more symptom in each syndrome, adjusting for patient demographics, comorbid diseases, physiologic data (laboratory data and vital signs within 12 hours of arrival, highest respiratory support, and vasopressor administration), history of penicillin or β-lactam allergies, and time from ED arrival to IV antibiotic delivery (hourly intervals until 6 hours, 6-9 hours, 9-12 hours, and 12-24 hours; further details are provided in the eMethods in Supplement 1).^17,42^

SEs were used to calculate Wald 95% CIs for each RR and AOR. We used Bonferroni adjustment for multiple hypotheses in crude comparisons of proportions (404 signs and symptoms; Fisher exact test), crude RRs (30 signs and symptoms), and AORs (7 syndromes). Spearman ρ was used to test correlations between ordinal variables. All analyses were performed from July 2023 to August 2025 using Python, version 3.11.7 (Python Software Foundation), and R, version 4.4.2 (R Project for Statistical Computing).

## Results

### Characteristics of Study Cohort

The cohort included 104 248 patients with possible infection.^17,42^ Patients had a median (IQR) age of 66 (52-78) years and included 50 106 females (48.1%) and 54 137 males (51.9%). A total of 23 619 patients (22.7%) had sepsis without shock, and 25 990 patients (24.9%) had septic shock.^17^

Admission notes were filed with a time of service within 24 hours of ED arrival for 94 913 of 104 248 patients (91.0%) (eFigure 1 in Supplement 1). Of these patients, 93 674 (98.7%) were labeled with 1 or more presenting signs and symptoms by the LLM method using a controlled vocabulary of 404 labels (eTable 2 in Supplement 1). Among patients with labeled signs and symptoms, 52 027 (55.5%) had 1 or more positive cultures collected within 72 hours of ED arrival, including 1903 (2.0%) with MRSA and 8617 (9.2%) with MDRGN organisms. The in-hospital mortality rate was 4464 of 93 674 (4.8%). The median (IQR) size of prompts processed by the LLM was 436 (348-564) tokens, with only 41 of 93 674 patients (0.04%) requiring truncation of the HPI to fit within the context window.

### Validation of the LLM Method

The LLM method achieved an accuracy of 99.3% (95% CI, 99.2%-99.3%), balanced accuracy of 84.6% (95% CI, 83.5%-85.8%), sensitivity of 69.7% (95% CI, 67.3%-72.0%), and specificity of 99.6% (95% CI, 99.6%-99.6%) against the primary physician medical record reviewer (Table 1). These metrics were similar to the performance of the primary vs secondary physician reviewers. Cohen κ for the LLM vs the primary physician reviewer was 0.69 (95% CI, 95% CI, 0.67-0.70), similar to the concordance between 2 physician reviewers (Cohen κ = 0.73; 95% CI, 0.70-0.76). The LLM’s positive predictive value per label was 68.4% (95% CI, 66.0%-70.7%) vs 76.6% (95% CI, 72.6%-80.2%) for one physician against another, while the negative predictive value was identical (99.6%; 95% CI, 99.6%-99.7%). The LLM’s performance characteristics were similar when comparing outputs against either physician’s labels.

**Table 1.** Validation of Labeling Presenting Signs and Symptoms With a Large Language Model vs Physician Medical Record Review

Metric	Value (95% CI), %		
	LLM vs primary physician reviewer	Primary physician vs secondary physician reviewers	LLM vs secondary physician reviewer
Method being validated	LLM	Primary physician	LLM
Gold standard or ground truth method	Primary physician	Secondary physician	Secondary physician
No. of admission notes compared	303	100	100
Total No. of possible labels	122 412	40 400	40 400
Accuracy	99.3 (99.2-99.3)	99.3 (99.2-99.4)	99.1 (99.0-99.2)
Balanced accuracy	84.6 (83.5-85.8)	84.7 (82.6-86.6)	81.9 (79.8-83.9)
Sensitivity	69.7 (67.3-72.0)	69.7 (65.6-73.4)	64.2 (60.1-68.2)
Specificity	99.6 (99.6-99.6)	99.7 (99.7-99.8)	99.6 (99.5-99.7)
F1 score	69.0 (67.1-70.9)	73.0 (69.9-76.1)	66.1 (62.8-69.5)
PPV	68.4 (66.0-70.7)	76.6 (72.6-80.2)	68.2 (64.0-72.1)
NPV	99.6 (99.6-99.7)	99.6 (99.5-99.7)	99.5 (99.5-99.6)
Cohen κ, No.	0.69 (0.67-0.70)	0.73 (0.70-0.76)	0.66 (0.62-0.69)

Abbreviations: LLM, large language model; NPV, negative predictive value; PPV, positive predictive value.

On all performance metrics, the LLM method exceeded the *ICD-10-CM* method in labeling admissions with presenting signs and symptoms (eTable 3 in Supplement 1). LLM performance characteristics did not improve with the use of multiple prompts to extract small sets of specific vocabulary terms instead of an open-ended prompt (eTable 4 in Supplement 1). The order of labels in the LLM’s output was significantly correlated with the order of appearance in the HPI text provided to the model (Spearman ρ* = *0.54; *P* < .001) (eFigure 2 in Supplement 1) but not with sign and symptom severity as measured by crude risk of death (eFigure 3 in Supplement 1).

### Clustering of Signs and Symptoms Into Syndromes

The 30 most prevalent signs and symptoms are summarized in Table 2. Among 93 674 patients with labeled signs and symptoms, the most frequently identified signs and symptoms were fever (36 286 [38.7%]), dyspnea (25 572 [27.3%]), and cough (24 018 [25.6%]). The prevalence of most signs and symptoms was comparable between all patients with labeled signs and symptoms and those with 1 or more positive cultures, although culture positivity was associated with higher rates of dysuria (5.9% [3087] vs 4.2% [3964]; *P* < .001) and urinary frequency (4.8% [2494] vs 3.5% [3321]; *P* < .001) and lower rates of redness (5.1% [2645] vs 8.5% [7928]; *P* < .001) and swelling (6.4% [3351] vs 9.4% [8817]; *P* < .001). Prevailing signs and symptoms differed for patients with MRSA-positive vs MDRGN-positive cultures. Patients with MRSA-positive culture were more likely to present with redness (15.8% [301] vs 3.4% [289]; *P* < .001) and swelling (16.9% [321] vs 4.0% [343]; *P* < .001) but less likely to present with dysuria (1.3% [24] vs 7.3% [629]; *P* < .001), urinary frequency (1.5% [28] vs 5.6% [486]; *P* < .001), and abdominal pain (12.4% [236] vs 24.2% [2088]; *P* < .001). Prevalence of all 404 signs and symptoms is provided in eTable 2 in Supplement 1.

**Table 2.** Thirty Most Common Presenting Signs and Symptoms and Prevalence in Patients With Any Positive Culture, Positive Cultures for Multidrug-Resistant Organisms, and In-Hospital Mortality

Sign or symptom^a^	Patients with labeled admission notes, No. (%)				
	All	≥1 Positive culture^b^	MRSA in ≥1 culture^b^	MDRGN organism in ≥1 culture^b^	In-hospital mortality
Total	93 764 (100)	52 027 (100)	1903 (100)	8617 (100)	4464 (100)
Fever	36 286 (38.7)	21 666 (41.6)^c^	815 (42.8)	3847 (44.6)	1025 (23.0)
Dyspnea	25 572 (27.3)	14 864 (28.6)^c^	579 (30.4)	1966 (22.8)^d^	2069 (46.3)
Cough	24 018 (25.6)	14 600 (28.1)^c^	534 (28.1)	1659 (19.3)^d^	1225 (27.4)
Abdominal pain	20 831 (22.2)	11 209 (21.5)	236 (12.4)	2088 (24.2)^d^	784 (17.6)
Pain	20 300 (21.7)	9491 (18.2)^c^	492 (25.9)	1447 (16.8)^d^	479 (10.7)
Nausea	20 036 (21.4)	10 881 (20.9)	252 (13.2)	1773 (20.6)^d^	648 (14.5)
Chills	19 923 (21.3)	11 725 (22.5)^c^	362 (19.0)	2094 (24.3)^d^	343 (7.7)
Fatigue	19 439 (20.8)	11 424 (22.0)^c^	268 (14.1)	1789 (20.8)^d^	1020 (22.8)
Vomiting	17 000 (18.1)	9307 (17.9)	227 (11.9)	1595 (18.5)^d^	641 (14.4)
Altered mental status	16 010 (17.1)	10 033 (19.3)^c^	341 (17.9)	2024 (23.5)^d^	1304 (29.2)
Diarrhea	11 941 (12.7)	6329 (12.2)	161 (8.5)	971 (11.3)	506 (11.3)
Chest pain	9503 (10.1)	5230 (10.1)	221 (11.6)	632 (7.3)^d^	459 (10.3)
Weakness	9212 (9.8)	5416 (10.4)	143 (7.5)	912 (10.6)	489 (11.0)
Swelling	8817 (9.4)	3351 (6.4)^c^	321 (16.9)	343 (4.0)^d^	188 (4.2)
Sputum changes	8080 (8.6)	5692 (10.9)^c^	269 (14.1)	752 (8.7)^d^	378 (8.5)
Redness	7928 (8.5)	2645 (5.1)^c^	301 (15.8)	289 (3.4)^d^	86 (1.9)
Headache	7335 (7.8)	3932 (7.6)	111 (5.8)	489 (5.7)	148 (3.3)
Poor appetite	7275 (7.8)	4274 (8.2)	105 (5.5)	670 (7.8)	367 (8.2)
Malaise	7207 (7.7)	4308 (8.3)	126 (6.6)	665 (7.7)	263 (5.9)
Back pain	5974 (6.4)	3582 (6.9)	151 (7.9)	530 (6.2)	215 (4.8)
Hypoxemia	4921 (5.3)	3111 (6.0)^c^	169 (8.9)	552 (6.4)	716 (16.0)
Hypotension	4093 (4.4)	2505 (4.8)	109 (5.7)	543 (6.3)	527 (11.8)
Dizziness	4009 (4.3)	2110 (4.1)	39 (2.0)	280 (3.2)	141 (3.2)
Dysuria	3964 (4.2)	3087 (5.9)^c^	24 (1.3)	629 (7.3)^d^	59 (1.3)
Rhinorrhea	3931 (4.2)	2308 (4.4)	57 (3.0)	246 (2.9)	123 (2.8)
Lightheadedness	3546 (3.8)	1868 (3.6)	36 (1.9)	241 (2.8)	146 (3.3)
Fall	3338 (3.6)	1931 (3.7)	44 (2.3)	304 (3.5)	206 (4.6)
Urinary frequency	3321 (3.5)	2494 (4.8)^c^	28 (1.5)	486 (5.6)^d^	55 (1.2)
Diaphoresis	3225 (3.4)	1779 (3.4)	72 (3.8)	299 (3.5)	92 (2.1)
Leg swelling	3100 (3.3)	1296 (2.5)^c^	50 (2.6)	155 (1.8)	160 (3.6)

Abbreviations: MDRGN, multidrug-resistant gram-negative; MRSA, methicillin-resistant *Staphylococcus aureus*.

^a^ All 404 signs and symptoms are presented with synonyms in eTable 2 in Supplement 1. For row-wise, instead of column-wise, percentages, see eTable 5 in Supplement 1.

^b^ Cultures collected within 72 hours of arrival to the emergency department from any of the following body sites: blood, urine, intra-abdominal fluid, pleural fluid, bronchial or bronchoalveolar lavage fluid, cerebrospinal fluid, pericardial fluid, retropharyngeal fluid, sputum, abscesses, deep tissue, joint spaces, kidney stones, and catheter tips.

^c^ Comparison of proportions with all patients labeled was significant at *P* < .001 (Fisher exact test with Bonferroni correction for 404 tests).

^d^ Comparison of proportions with patients with MRSA-positive cultures was significant at *P* < .001 (Fisher exact test with Bonferroni correction for 404 tests).

**Figure 1.**
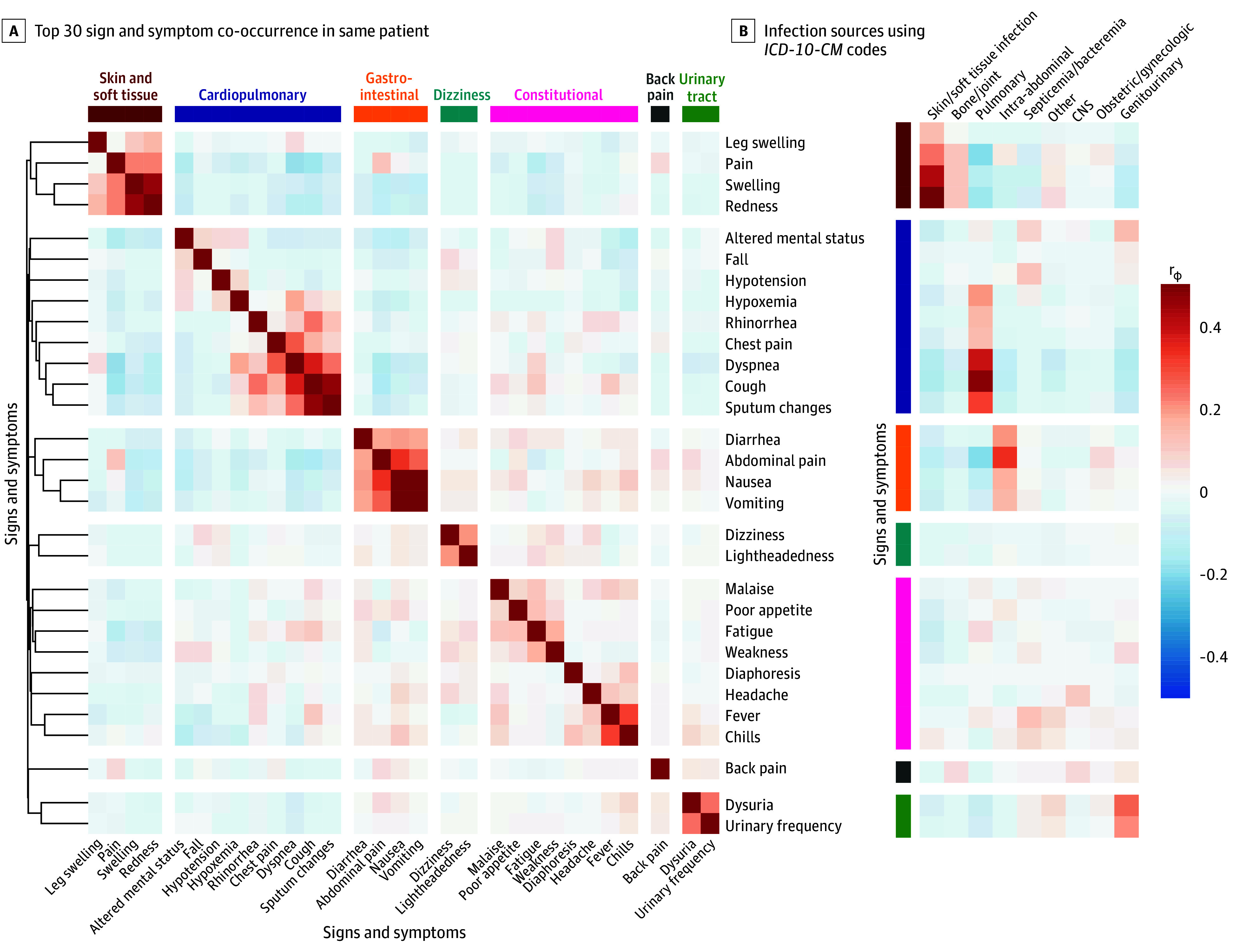
Clustering of Signs and Symptoms in Patients With Possible Infection and Syndromes Correlating With Infection Sources Derived From Discharge Diagnosis Codes Heatmap values correspond to Pearson correlation coefficients (*r*_Φ_ scale). Single-linkage hierarchical clustering was performed (dendrogram at left) followed by a tree cut into 7 syndromes. CNS indicates central nervous system; *ICD-10-CM, International Statistical Classification of Diseases, Tenth Revision, Clinical Modification*.

Hierarchical clustering of the 30 most common signs and symptoms by co-occurrence (Figure 1A) produced 7 syndromes corresponding to 4 common sites of infection (skin and soft tissue, cardiopulmonary, gastrointestinal, and urinary tract) as well as 3 nonspecific symptom clusters (dizziness, back pain, and constitutional). Clusters remained largely stable across different distance metrics and linkage criteria (eFigures 4 and 5 in Supplement 1). The 4 site-specific syndromes were directly correlated with *ICD-10-CM* discharge diagnosis codes that corresponded to infections at those sites (Figure 1B).

### Associations of Signs and Symptoms With Risk of MRSA or MDRGN Infection

**Figure 2.**
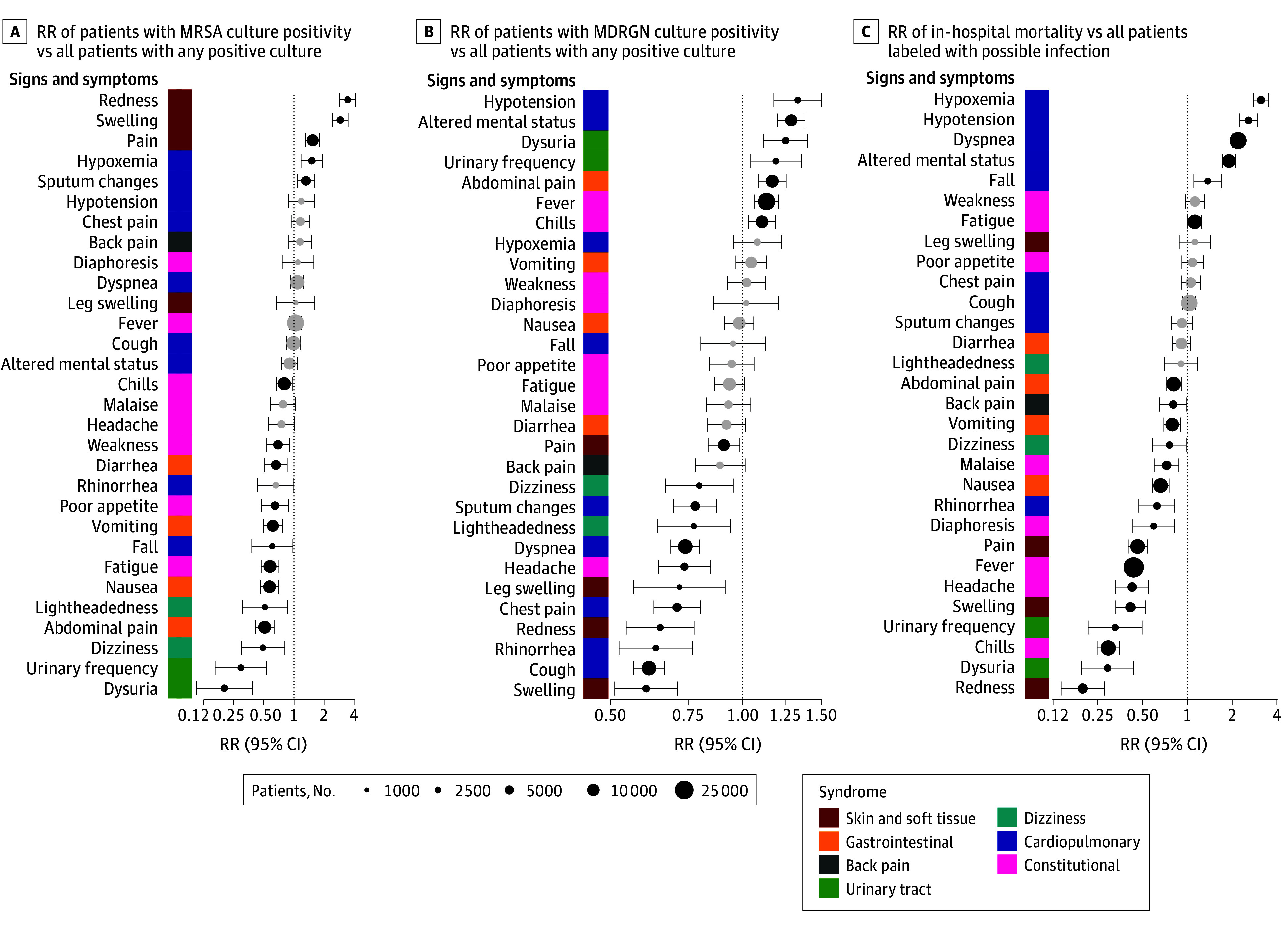
Crude Relative Risks (RRs) of Culture Positivity for Methicillin-Resistant *Staphylococcus aureus* (MRSA), Culture Positivity for Multidrug-Resistant Gram-Negative (MDRGN) Organisms, and In-Hospital Mortality for 30 Signs and Symptoms Wald tests with Bonferroni correction were used to construct 95% CIs (error bars). RRs not significantly different from 1 at *P* < .05 are shaded light gray. The x-axis is logarithmically scaled.

**Figure 3.**
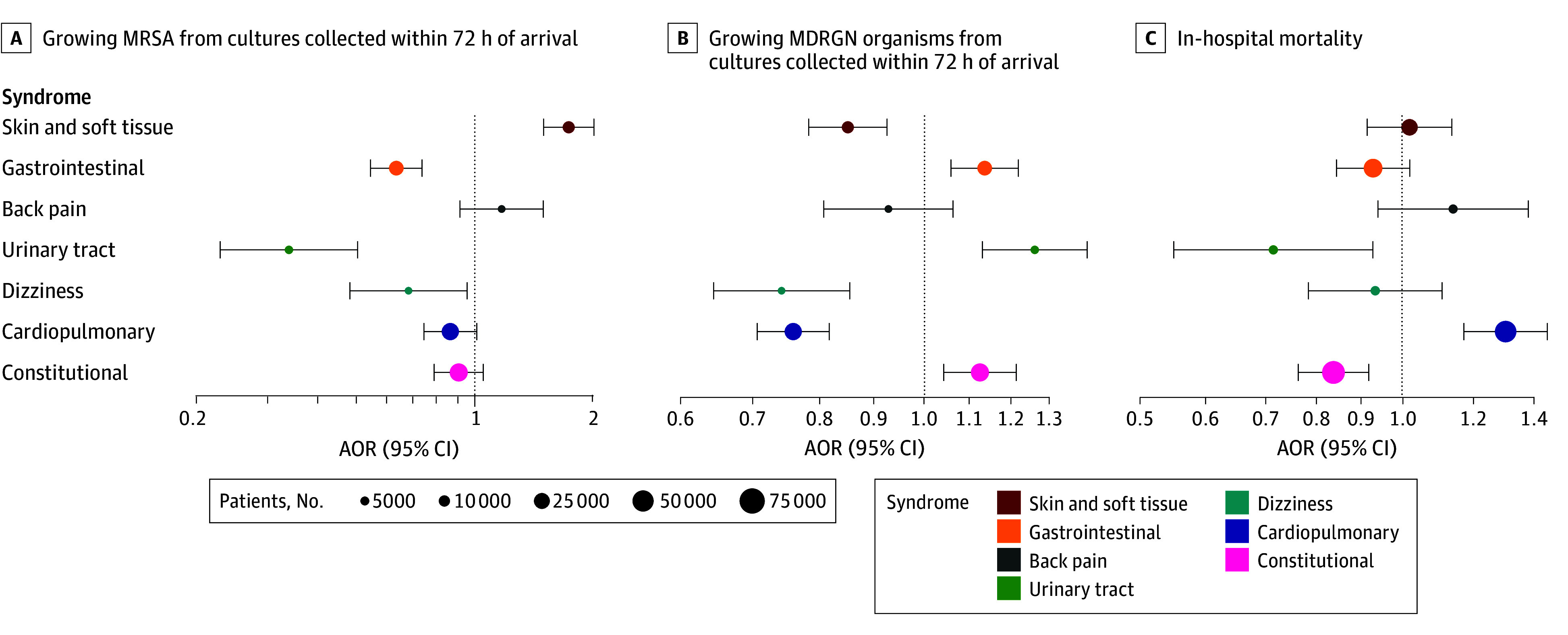
Adjusted Odds Ratios (AORs) for Culture Positivity for Methicillin-Resistant *Staphylococcus aureus* (MRSA), Culture Positivity for Multidrug-Resistant Gram-Negative (MDRGN) Organisms, and In-Hospital Mortality for Each Syndrome Multivariable logistic regression with adjustment for confounders was used to model associations between patients having 1 or more sign and symptom in each of the 7 syndromes and odds of 3 outcomes (A, B, and C). AORs were drawn with Bonferroni-adjusted 95% CIs (error bars). The x-axis is logarithmically scaled. Circle sizes are scaled to the number of patients assigned to each syndrome.

Among patients with positive cultures, crude RRs between signs and symptoms and MRSA culture positivity were lowest among patients with urinary tract and gastrointestinal symptoms and highest for skin and soft tissue symptoms (Figure 2A). RRs ranged from 0.20 (95% CI, 0.11-0.39) for dysuria to 3.51 (95% CI, 2.91-4.23) for redness (eTable 6 in Supplement 1). After clustering by syndrome and adjusting for confounders (Figure 3A; eTable 9 in Supplement 1), skin and soft tissue symptoms were directly associated with MRSA (AOR, 1.73; 95% CI, 1.49-2.00) whereas urinary tract symptoms (AOR, 0.34; 95% CI, 0.22-0.50), gastrointestinal symptoms (AOR, 0.63; 95% CI, 0.54-0.73), and dizziness (AOR, 0.68; 95% CI, 0.48-0.95) were inversely associated with MRSA.

By contrast, crude RRs between signs and symptoms and MDRGN organisms (Figure 2B) were lowest in patients with skin and soft tissue and respiratory symptoms (lowest RR for swelling, 0.60; 95% CI, 0.51-0.71) and highest in patients with hypotension, altered mental status, and urinary tract symptoms (highest RR for hypotension, 1.33; 95% CI, 1.17-1.50) (eTable 7 in Supplement 1). After adjusting for confounders (Figure 3B; eTable 9 in Supplement 1), several syndromes were inversely associated with MDRGN organisms, including skin and soft tissue symptoms (AOR, 0.85; 95% CI, 0.79-0.93), cardiopulmonary symptoms (AOR, 0.76; 95% CI, 0.71-0.82), and dizziness (AOR, 0.74; 95% CI, 0.64-0.86). Conversely, urinary tract (AOR, 1.26; 95% CI, 1.13-1.41), gastrointestinal (AOR, 1.14; 95% CI, 1.06-1.22), and constitutional symptoms (AOR, 1.12; 95% CI, 1.04-1.21) were directly associated with MDRGN organisms.

### Associations of Signs and Symptoms With Mortality

Crude RRs between signs and symptoms and in-hospital mortality were lowest for patients with skin and soft tissue and urinary tract symptoms and highest for patients with cardiopulmonary symptoms, ranging from 0.19 (95% CI, 0.14-0.27) for redness to 3.17 (95% CI, 2.82-3.57) for hypoxemia (Figure 2C; eTable 8 in Supplement 1). After clustering by syndrome and adjusting for confounders (Figure 3C; eTable 9 in Supplement 1), cardiopulmonary symptoms were associated with higher mortality (AOR, 1.30; 95% CI, 1.17-1.45), and conversely, urinary tract symptoms (AOR, 0.71; 95% CI, 0.55-0.92), and constitutional symptoms (AOR, 0.83; 95% CI, 0.76-0.91) were associated with lower mortality.

## Discussion

We developed and validated a method using an LLM to extract presenting signs and symptoms from clinical notes for a large cohort of patients with possible infection. To our knowledge, this study was the first to analyze presenting sign and symptom data in hospitalized patients at this scale. The LLM-generated labels achieved high accuracy compared with the physician-generated labels, enabling the efficient labeling of 93 674 admission notes using a controlled vocabulary of 404 signs and symptoms. Hierarchical clustering of the most prevalent signs and symptoms produced recognizable syndromes corresponding to common infection sources. RRs of MRSA, MDRGN organisms, and in-hospital mortality differed substantially based on patients’ presenting signs and symptoms.

Our findings have several implications for sepsis and clinical research. First, they demonstrate the feasibility of using LLMs to extract complex data from unstructured clinical text at a scale not possible with manual medical record review. Notwithstanding the concerns about LLMs’ interpretability and potential biases, we found that a publicly downloadable LLM achieved performance on par with performance of physicians for labeling signs and symptoms when given plain English instructions and mapping outputs to a controlled vocabulary. Local use of a downloadable LLM enabled efficient and secure labeling of clinical notes containing protected health information. By enabling new population-scale analyses of clinically significant patient-level details in clinical notes, including symptoms, temporality, outside hospital courses, and other health care exposures, LLMs can advance the scope and quality of clinical epidemiologic research. LLMs may also accelerate similar tasks requiring medical record abstraction, including clinical trial enrollment,^43^ hospital quality metrics,^44^ ED triage,^45^ and handoff documentation.^46^

Second, the symptom clusters identified in this study provide a more intuitive basis for categorizing patients with sepsis beyond organ dysfunction or biomarker criteria. The self-organization of LLM-generated signs and symptoms into syndromes that mirror common infection sources and correlate with discharge diagnoses supports the construct validity of the LLM method. Unlike discharge diagnosis codes, signs and symptoms more accurately reflect the ambiguity of initial patient presentations, considering that 3 of the identified syndromes (dizziness, constitutional, and back pain) were not specific to an infection source. When compared with the *ICD-10-CM* method of identifying presenting signs and symptoms using codes from professional billing charges up to the date of admission, the LLM method exceeded the *ICD-10-CM* method in sensitivity, specificity, and precision.

Third, the observed differential risks of MRSA, MDRGN organisms, and in-hospital mortality across symptoms highlight these data’s importance to understanding the heterogeneity of patients with possible sepsis. For example, the fact that certain symptoms were associated with lower risk for specific multidrug-resistant organisms (urinary tract symptoms for MRSA; cellulitis symptoms for MDRGN organisms) suggests that empiric antibiotic guidelines for sepsis could likely be tailored further using presenting symptoms. The associations between decreased mortality risk and urinary or constitutional symptoms suggest that broadly aggressive sepsis treatment protocols could unintentionally incur a less favorable benefit-to-risk ratio in these subcohorts. Additionally, the association between constitutional symptoms (most commonly fever and chills in this cohort) and decreased mortality aligns with prior work documenting that explicit infectious symptoms in sepsis cohorts are associated with earlier treatment and better outcomes.^20,21^ Future risk prediction and causal inference models for sepsis should incorporate symptoms and assess the potential benefits and risks of adding symptom-based criteria to consensus guidelines on empiric antibiotic timing and selection.

### Limitations

This study has several limitations. First, although we validated the LLM method against physician medical record review, both are limited by the quality of symptom documentation in clinical notes. Certain symptoms in patients who are unable to communicate (eg, critically ill patients) may have been systematically excluded. Second, symptoms are subjective, and disagreement between clinicians on the connotation of terms and what can be inferred from context creates variability in labeling HPIs (as measured in this study) and subjectivity in the construction of sign and symptom vocabularies. Third, the black-box nature of LLMs limited our ability to understand the reasons for discrepancies between LLM-generated labels and physician-generated labels. Fourth, although we adjusted associations against a comprehensive set of confounders, residual confounding cannot be excluded. Finally, our data were obtained from 1 regional US health system, although our use of a publicly available LLM without fine-tuning suggests that the method likely generalizes to other settings.

## Conclusions

In this cohort study of hospitalized patients with possible infection, an LLM accurately extracted presenting signs and symptoms from admission notes and identified data-driven symptom-based syndromes that were differentially correlated with infection sources, risk of infection by multidrug-resistant pathogens, and in-hospital mortality. Further research is warranted to evaluate the additive value of large-scale sign-and-symptom data as mediators or confounders in models of antibiotic choice, effectiveness, and outcomes in patients with possible sepsis.

## Notes

Supplement 1.
eMethods.
**eFigure 1.** Study Flow Diagram **eFigure 2.** Correlation Analysis Between Sign/Symptom Position in LLM Output and Order of Appearance in the Corresponding HPI **eFigure 3.** Correlation Analysis Between Sign/Symptom Position in LLM Output and Sign/Symptom Severity as Measured by Crude Mortality Risk **eFigure 4.** Clustering of Signs/Symptoms in Patients With Possible Infection Using Pairwise Distance (1 – *r*_Φ_) **eFigure 5.** Clustering of Signs/Symptoms in Patients With Possible Infection Using Pairwise Jaccard Distance **eTable 1.** Mapping of the 30 Most Prevalent Signs/Symptoms in the Study Cohort to Equivalent ICD-10-CM Code Ranges **eTable 2.** Prevalence of All Signs and Symptoms in the Controlled Vocabulary **eTable 3.** Comparative Validation of LLM vs ICD-10-CM Strategies for Extracting the Top 30 Presenting Signs/Symptoms Against Gold Standard Human Chart Review **eTable 4.** Comparative Validation of the Original LLM Strategy vs an Alternative LLM Strategy of Prompting for Five Signs/Symptoms at a Time Against Gold Standard Human Chart Review **eTable 5.** Thirty Most Prevalent Signs/Symptoms in Possible Infection Admissions and Proportions for Each Sign/Symptom of Culture Positivity and Mortality **eTable 6.** Crude Relative Risk of MRSA Culture Positivity for Each of the Top 30 Signs/Symptoms **eTable 7.** Crude Relative Risk of MDR Gram-Negative Culture Positivity for Each of the Top 30 Signs/Symptoms **eTable 8.** Crude Relative Risk of In-Hospital Mortality for Each of the Top 30 Signs/Symptoms **eTable 9.** Adjusted Odds Ratios for Culture Positivity for MRSA, Culture Positivity for MDRGN Organisms, and In-Hospital Mortality for Each Syndrome
eReferences

Supplement 2.
Data Sharing Statement
