# Supplementary material for: Syndromic Analysis of Sepsis Cohorts Using Large Language Models
Source: JAMA Netw Open. 2025 Oct 24;8(10):e2539267. doi: 10.1001/jamanetworkopen.2025.39267 (PMC12552932; doi:10.1001/jamanetworkopen.2025.39267)
Supplement: Supplement 1. — eMethods. eFigure 1. Study Flow Diagram eFigure 2. Correlation Analysis Between Sign/Symptom Position in LLM Output and Order of Appearance in the Corresponding HPI eFigure 3. Correlation Analysis Between Sign/Symptom Position in LLM Output and Sign/Symptom Severity as Measured by Crude Mortality Risk eFigure 4. Clustering of Signs/Symptoms in Patients With Possible Infection Using Pairwise Distance (1 – rΦ) eFigure 5. Clustering of Signs/Symptoms in Patients With Possible Infection Using Pairwise Jaccard Distance eTable 1. Mapping of the 30 Most Prevalent Signs/Symptoms in the Study Cohort to Equivalent ICD-10-CM Code Ranges eTable 2. Prevalence of All Signs and Symptoms in the Controlled Vocabulary eTable 3. Comparative Validation of LLM vs ICD-10-CM Strategies for Extracting the Top 30 Presenting Signs/Symptoms Against Gold Standard Human Chart Review eTable 4. Comparative Validation of the Original LLM Strategy vs an Alternative LLM Strategy of Prompting for Five Signs/Symptoms at a Time Against Gold Standard Human Chart Review eTable 5. Thirty Most Prevalent Signs/Symptoms in Possible Infection Admissions and Proportions for Each Sign/Symptom of Culture Positivity and Mortality eTable 6. Crude Relative Risk of MRSA Culture Positivity for Each of the Top 30 Signs/Symptoms eTable 7. Crude Relative Risk of MDR Gram-Negative Culture Positivity for Each of the Top 30 Signs/Symptoms eTable 8. Crude Relative Risk of In-Hospital Mortality for Each of the Top 30 Signs/Symptoms eTable 9. Adjusted Odds Ratios for Culture Positivity for MRSA, Culture Positivity for MDRGN Organisms, and In-Hospital Mortality for Each Syndrome eReferences [file jamanetwopen-e2539267-s001.pdf]

Supplemental Online Content

Pak TR, Kanjilal S, McKenna CS, Hoffner-Heinike A, Rhee C, Klompas M. Syndromic analysis of sepsis cohorts using large language models. *JAMA Netw Open*. 2025;8(10):e2539267. doi:10.1001/jamanetworkopen.2025.39267

eMethods
eFigure 1. Study Flow Diagram
eFigure 2. Correlation Analysis Between Sign/Symptom Position in LLM Output and Order of Appearance in the Corresponding HPI
eFigure 3. Correlation Analysis Between Sign/Symptom Position in LLM Output and Sign/Symptom Severity as Measured by Crude Mortality Risk
eFigure 4. Clustering of Signs/Symptoms in Patients With Possible Infection Using Pairwise Distance ($1 - r_{\Phi}$)
eFigure 5. Clustering of Signs/Symptoms in Patients With Possible Infection Using Pairwise Jaccard Distance
eTable 1. Mapping of the 30 Most Prevalent Signs/Symptoms in the Study Cohort to Equivalent ICD-10-CM Code Ranges
eTable 2. Prevalence of All Signs and Symptoms in the Controlled Vocabulary
eTable 3. Comparative Validation of LLM vs ICD-10-CM Strategies for Extracting the Top 30 Presenting Signs/Symptoms Against Gold Standard Human Chart Review
eTable 4. Comparative Validation of the Original LLM Strategy vs an Alternative LLM Strategy of Prompting for Five Signs/Symptoms at a Time Against Gold Standard Human Chart Review
eTable 5. Thirty Most Prevalent Signs/Symptoms in Possible Infection Admissions and Proportions for Each Sign/Symptom of Culture Positivity and Mortality
eTable 6. Crude Relative Risk of MRSA Culture Positivity for Each of the Top 30 Signs/Symptoms
eTable 7. Crude Relative Risk of MDR Gram-Negative Culture Positivity for Each of the Top 30 Signs/Symptoms
eTable 8. Crude Relative Risk of In-Hospital Mortality for Each of the Top 30 Signs/Symptoms
eTable 9. Adjusted Odds Ratios for Culture Positivity for MRSA, Culture Positivity for MDRGN Organisms, and In-Hospital Mortality for Each Syndrome
eReferences

This supplemental material has been provided by the authors to give readers additional information about their work.

© 2025 Pak TR et al. *JAMA Network Open*

eMethods

Detection of History of Present Illness Sections

After reviewing 100 random admitting History and Physical notes for common text that delimited the start and end of the History of Present Illness (HPI) section, we constructed the following regular expressions for extracting HPI sections, implemented in the Python language (version 3.11):

```
import re

START_REGEX = re.compile(r"""
\b(
    HPI
    | (History|Central[ ]Elements)[ ]of[ ](the[ ])?
        (Present(ing)?[ ]Illness|Traumatic[ ]Injury)
    | Brief[ ]summary
)\b(
    :
    | \s+Comments:\s*
    | \s+HPI\b
    | \s+History[ ]of[ ](the[ ])?Present[ ]Illness
    | \s+Chief[ ]Complaint:[^\n]*
)*
""", re.VERBOSE | re.IGNORECASE)

END_REGEX = re.compile(r"""
(
    \n (
        History[ ]reviewed.[ ]+No[ ]pertinent[ ]past[ ]medical[ ]history.
    )
    | \n (
        Electronic[ ]Medical[ ]Records[ ]Reviewed
        | History[ ]provided[ ]by
        | (E[DWR]|Emergency[ ](Department|Room))[ ]Course
        | Current[ ]Assessment
        | Historical[ ]features
        | Focused[ ]COVID[ ]History
        | (ED[ ]Triage[ ])? (Vitals|Vital[ ]Signs)
        | (Plan|A[/&]P)
        | (Estimated[ ]Date[ ]of[ ]Delivery|EDD)
        | (Relevant|Pertinent[ ])? (Home[ ])? Medications
        | (Relevant|Pertinent[ ])? (Physical[ ])? Exam
        | Quality[ ]Bundle
    ) [:\n]
    | \n (
        Review[ ]of[ ]Systems
        | ROS
        | Past[ ](Medical[ ])?(History|Hx)
        | Patient[ ]Active[ ]Problem[ ]List
        | Medical (([/\s]+|[ ]+(\band\b|&)[ ]+)Surgical)? [ ](History|Hx)
        | PMH
        | (In[ ]|On[ ]arrival[ ](to|at)[ ]) (the[ ])? (\w+[ ])?
            (E[DWR]|Emergency[ ](Room|Department))
        | Data reviewed
        | ((Impression|Assessment)[ ]and[ ]) Plan
    ) \b
)
""", re.VERBOSE | re.IGNORECASE)
```

© 2025 Pak TR et al. *JAMA Network Open*

eMethods (cont.)

Alternative Prompting Strategy With Multiple Prompts Asking About Specific Signs/Symptoms

To see if performance would change under a different prompting strategy that asked the large language model (LLM) to evaluate for presence/absence of up to five specific signs/symptoms at a time, we implemented the following prompt template:

> **System:** You are a clinical researcher that reads medical charts and answers questions about them. Use only the information in the text provided to answer the question. After you provide an answer, you immediately stop talking.
>
> **User:** Read the following patient history and figure out which of the following presenting symptoms are reported for this patient: **[Five symptoms inserted here]**. Include only symptoms present now or reported for the days to weeks leading up to admission. Ignore any symptoms from past medical history or prior hospital admissions. Give your answer as a JSON object with the symptoms as the keys and true/false as the values. For example, if all symptoms are absent, the answer would be: **[Example JSON inserted here]**
>
> Now, here is the patient history: **[HPI contents inserted here]**
>
> **Assistant: …**

This prompt would be repeated for each group of up to five terms to query the full controlled vocabulary of 404 terms (eTable 2).

Culture Data and Multidrug-Resistant Gram-negative Organisms

When collecting all culture data from within 72h of arrival to the emergency department (ED), permissible culture sites included blood, urine, body fluid (intra-abdominal, pleural, bronchial, bronchoalveolar lavage, cerebrospinal, pericardial, or retropharyngeal), sputum, abscesses, deep tissue, joint spaces, renal stones, and catheter tips. Cultures drawn for infection control purposes (e.g., MRSA nasal swab) were excluded. Multidrug-resistant gram-negative (MDRGN) organisms were defined as gram-negative organisms intrinsically resistant to ceftriaxone (*Stenotrophomonas* and *Pseudomonas*), *Acinetobacter* resistant to ampicillin-sulbactam, clinically significant AmpC-producing *Enterobacterales* (*Enterobacter cloacae*, *Citrobacter freundii*, and *Klebsiella aerogenes* isolates),[1] ESBL-producing *Enterobacterales* (defined by ceftriaxone non-susceptibility or detection of ESBL genes on PCR), and *Enterobacterales* resistant to carbapenems.

Confounders Included in Multivariable Logistic Regression

Multivariable logistic regression models were adjusted for a comprehensive set of confounders collected from structured fields in the electronic health record, including: age, race/ethnicity recorded during ED registration, sex recorded during registration, English or non-English language preference, median income of the patient's home ZIP code, admitting hospital type (academic vs community), admission from an outside facility, insurance type, arrival by ambulance, year of the study (as a categorical variable), discharge from a hospital within the prior 90 days, presence of a β-lactam allergy, presence of any antibiotic allergy, presence of selected comorbidities using Elixhauser categorizations of prior discharge diagnosis codes (chronic lung diseases, diabetes, heart failure, liver disease, renal disease, leukemia, lymphoma, and solid tumors with and without metastases), the calculated Elixhauser comorbidity index,[2] presenting laboratory data (platelets, hematocrit, WBC, lactate, total bilirubin, aspartate aminotransferase, albumin, sodium, glucose, creatinine, and anion gap), pre-arrival intubation, body mass index, highest respiratory support, use of vasopressors within 12h of ED arrival, time from ED arrival to IV antibiotic delivery, and the first recorded temperature, heart rate, respiratory rate, and systolic blood pressure. Additional details and population-level summaries of these data in our cohort are available in prior studies.[3,4]

eFigure 1. Study flow diagram

Abbreviations: H&P, history and physical; HPI, history of presenting illness; LLM, large language model; MDRGN, multidrug-resistant gram-negative organism; MGB, Mass General Brigham; MRSA, methicillin-resistant *Staphylococcus aureus*; MDRGN, multidrug-resistant gram-negative organism.

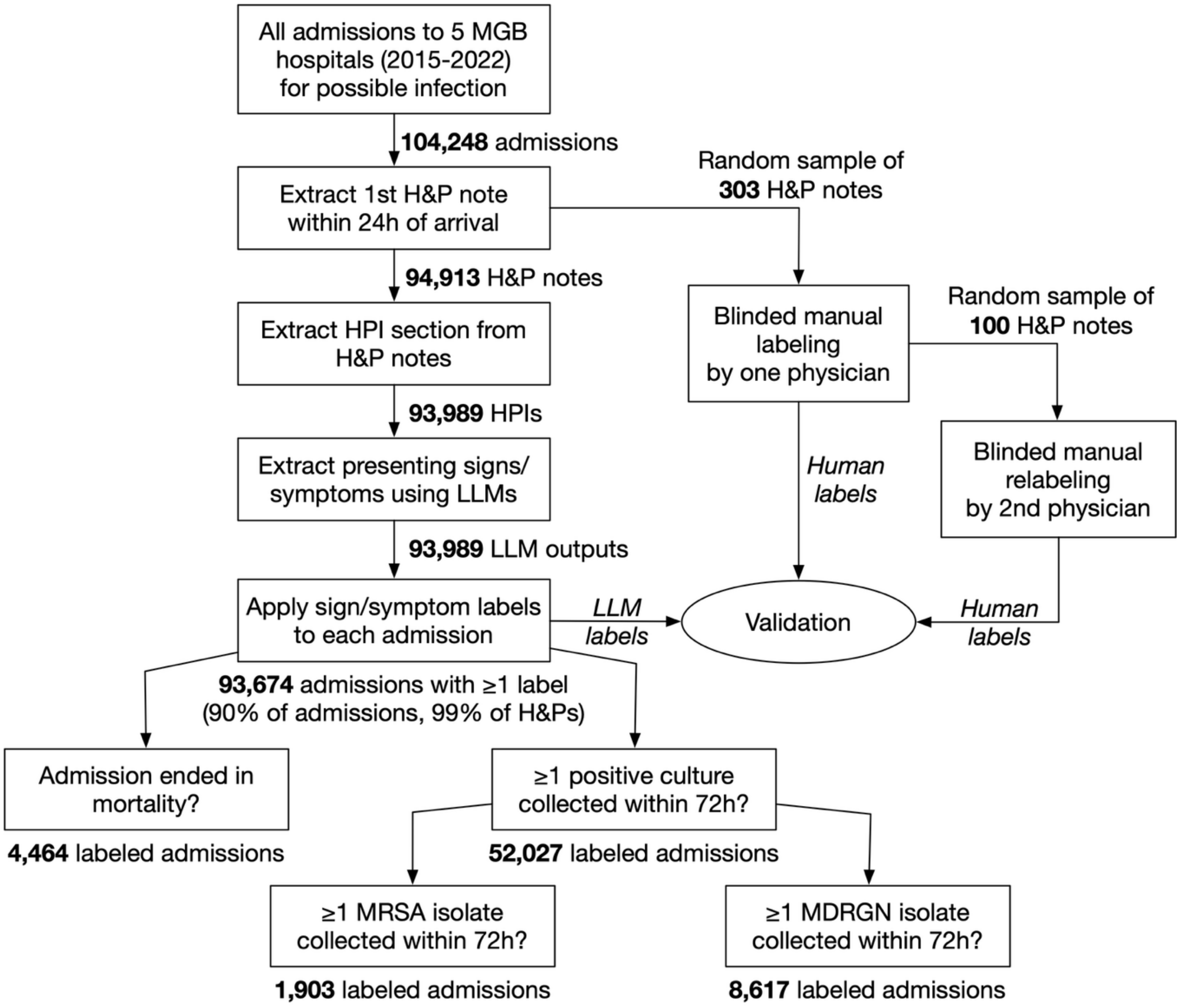

© 2025 Pak TR et al. *JAMA Network Open*

eFigure 2. Correlation analysis between sign/symptom position in LLM output and order of appearance in the corresponding HPI.

All 404 signs/symptoms are included, with 437,389 labels (out of 481,098 total) matched to corresponding orders of appearance in the HPI and depicted as individual points. Points are randomly jittered within each unit square and transparently shaded to reduce overplotting.

Abbreviations: HPI, history of present illness; LLM, large language model.

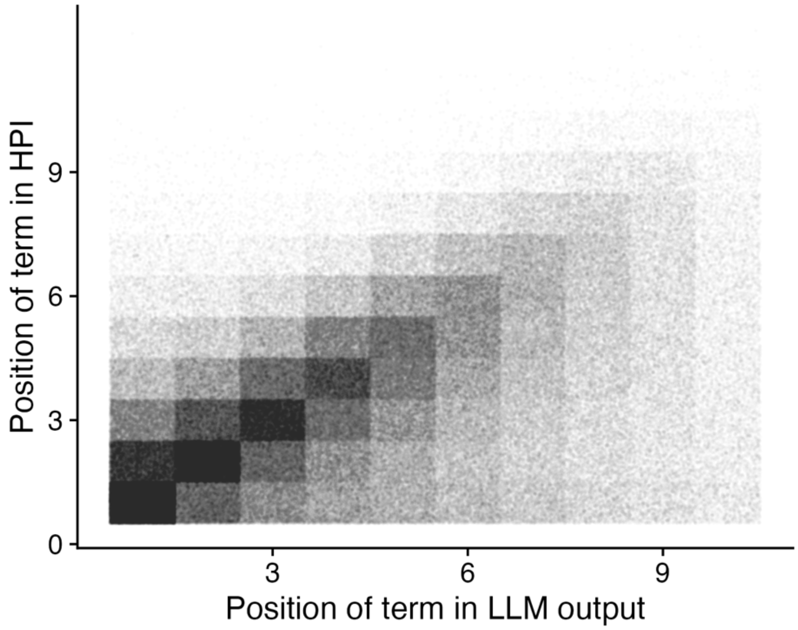

© 2025 Pak TR et al. *JAMA Network Open*

eFigure 3. Correlation analysis between sign/symptom position in LLM output and sign/symptom severity as measured by crude mortality risk.

The top 30 signs/symptoms are included. The Y axis is logarithmically scaled and depicts point estimates for crude relative risk of mortality for that sign/symptom (see Figure 2C). Points are scaled to the number of patients labeled with the sign/symptom (“count” in Legend). Pearson *r* = -0.22 (95% CI, -0.54 to +0.15).

Abbreviations: CI, confidence interval; LLM, large language model; RR, relative risk.

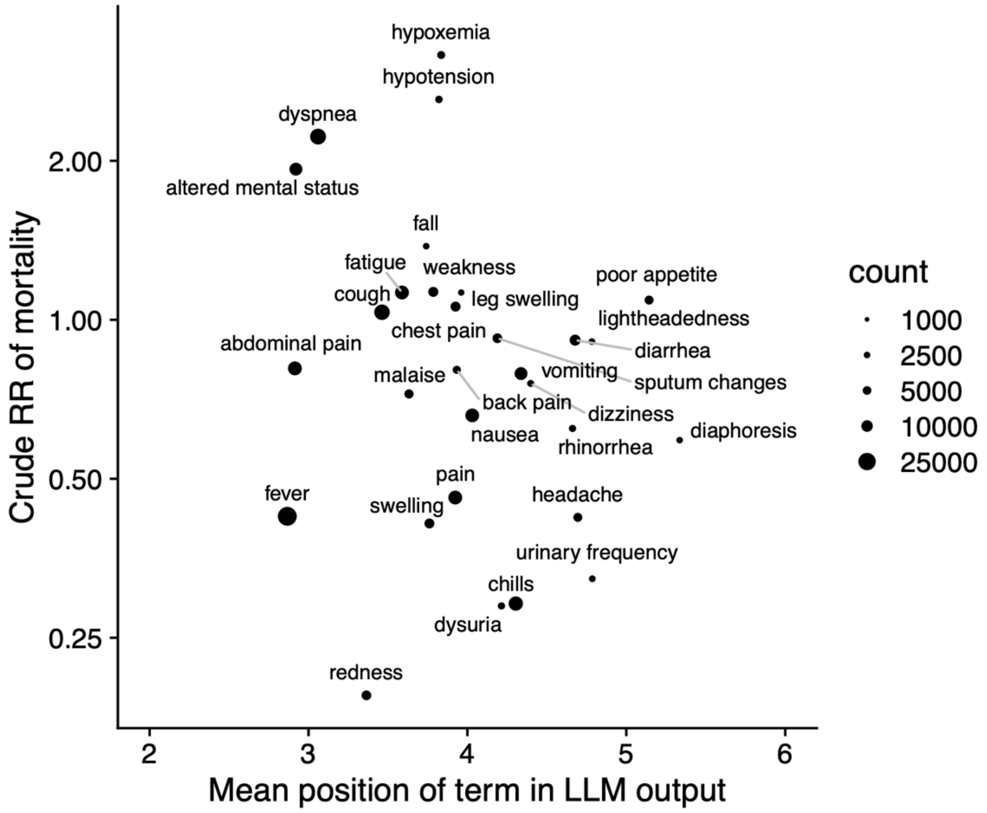

© 2025 Pak TR et al. *JAMA Network Open*

eFigure 4. Clustering of signs/symptoms in patients with possible infection using pairwise distance (1 – r_Φ).

Co-occurrence for the top 30 signs/symptoms in the same patient is plotted as a heatmap, with values corresponding to Pearson correlation coefficients (identical to r_Φ coefficients; red-blue scale, right-hand side). Hierarchical clustering was performed using a pairwise distance metric of 1 – r_Φ (dendrogram at left) and Ward's criteria for linkage[5] followed by a tree cut into 7 clusters. A comparison between these clusters and the original clusters (in Figure 1A) is plotted as colored bars along the top of the heatmap (see "original cluster," Legend).

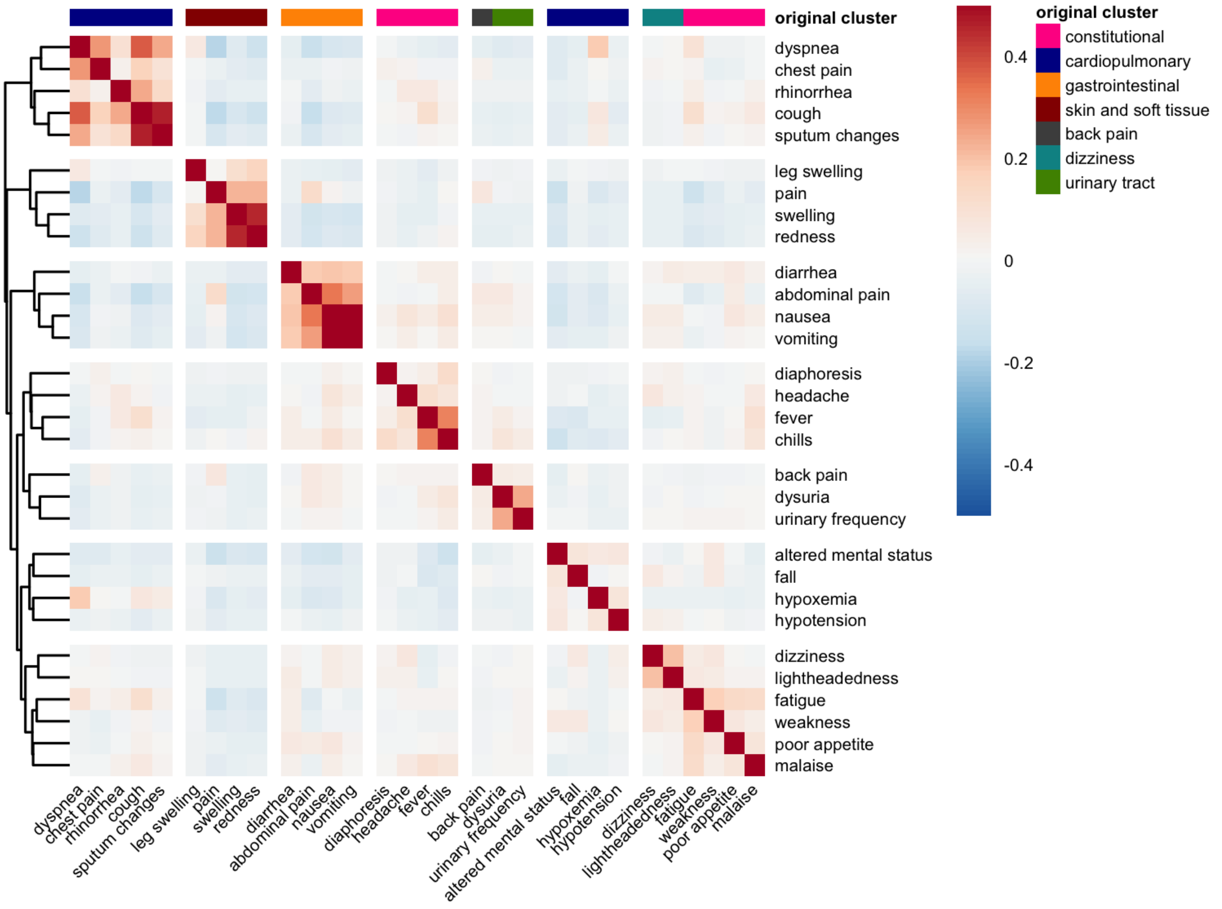

© 2025 Pak TR et al. *JAMA Network Open*

eFigure 5. Clustering of signs/symptoms in patients with possible infection using pairwise Jaccard distance.

Pairwise Jaccard distances between the top 30 signs/symptoms are plotted as a heatmap (colored scale, right-hand edge). Hierarchical clustering was performed using these distances (dendrogram at left) and Ward's criteria for linkage[5] followed by a tree cut into 7 clusters. A comparison between these clusters and the original clusters (in Figure 1A) is plotted as colored bars along the top of the heatmap (see "original cluster," Legend).

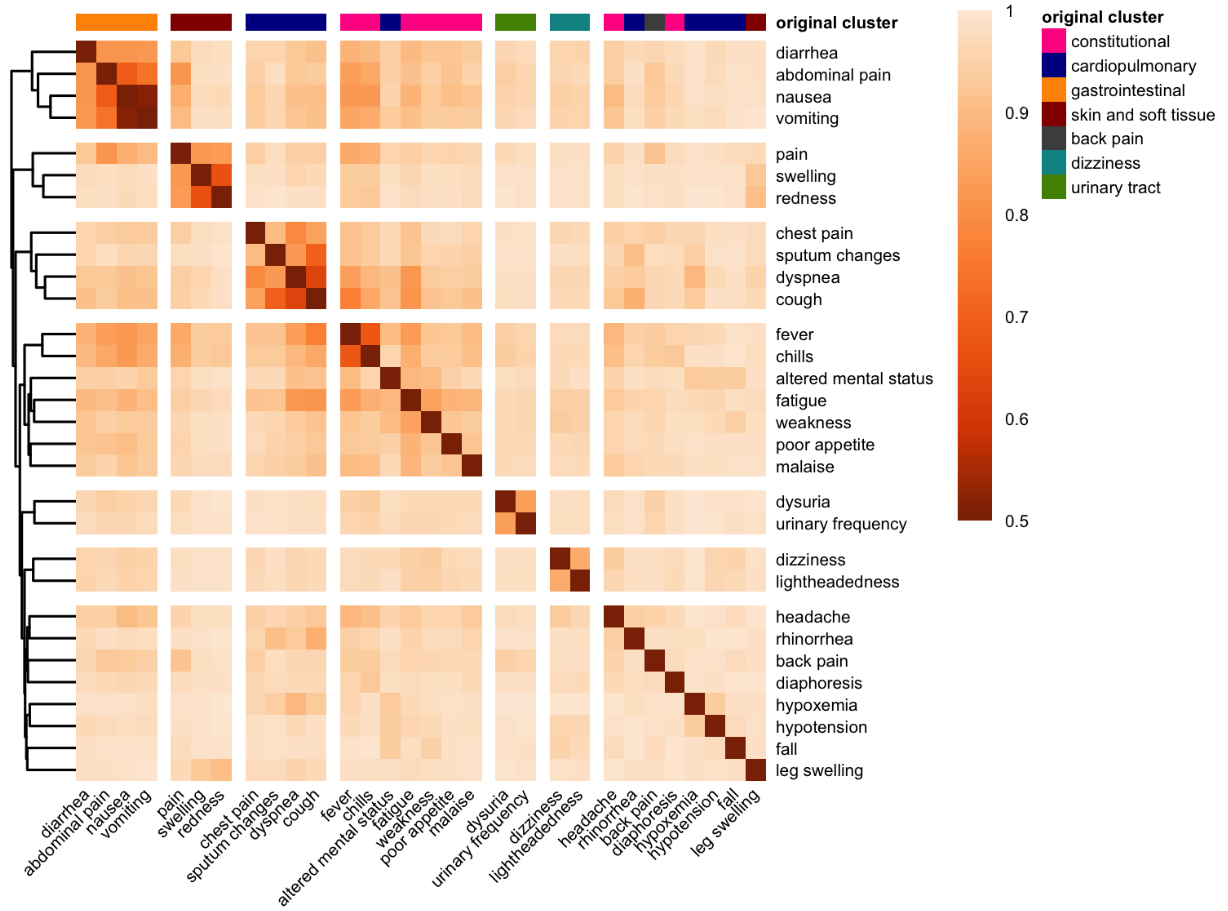

© 2025 Pak TR et al. *JAMA Network Open*

eTable 1. Mapping of the 30 most prevalent signs/symptoms in the study cohort to equivalent ICD-10-CM code ranges.

Sign or symptom	ICD-10-CM range prefixes[a]
Fever	R50.9
Dyspnea	R06.0
Cough	R05
Abdominal pain	R10
Pain	R52
Nausea	R11.0
Chills	R68.83
Fatigue	R53.8
Vomiting	R11
Altered mental status	R40 R41
Diarrhea	R19.7
Chest pain	R07.1 R07.2 R07.8 R07.9
Weakness	R53.1
Swelling	R60
Sputum changes	R09.3
Redness	R21 R23.2 L53.8 L53.9
Headache	R51
Poor appetite	R63.0 R63.3 R63.4 R63.6
Malaise	R53.81
Back pain	M54
Hypoxemia	R09.02
Hypotension	R03.1 I95
Dizziness	R42
Dysuria	R30.0 R30.9
Rhinorrhea	R09.81
Lightheadedness	R42
Fall	W0 W1
Urinary frequency	R35
Diaphoresis	R61
Leg swelling	R22.4

Abbreviations: ICD-10-CM, International Classification of Diseases, Tenth Revision, Clinical Modification.

[a] Ranges are given as space-delimited code prefixes, such that “W0” corresponds to all codes starting with W00-W09 and each specific code within, e.g., “W09.0XXS”.

© 2025 Pak TR et al. *JAMA Network Open*

eTable 2. Prevalence of all signs and symptoms in the controlled vocabulary.

	Patients with labeled admission notes, No. (%)					
	All	And with ≥1 positive culture[a]	And with MRSA in ≥1 culture[a]	And with MDRGN in ≥1 culture[a]	And with in-hospital mortality	All synonyms[b]
**Total**	93,674 (100)	52,027 (100)	1,903 (100)	8,617 (100)	4,464 (100)	
**By sign and symptom**						
fever	36,286 (38.7)	21,666 (41.6)	815 (42.8)	3,847 (44.6)	1,025 (23.0)	101 fever | 101f | 102 f | 102 fever | 102f | 103 fever | 103f | 104f | febrile | febrile neutropenia | fever tmax | feverish | fevers | grade fever | intermittent fevers | low grade fever | low grade fevers | subjective fever | subjective fevers
dyspnea	25,572 (27.3)	14,864 (28.6)	579 (30.4)	1,966 (22.8)	2,069 (46.3)	difficulty breathing | doe | exertional dyspnea | increased dyspnea | mild dyspnea | progressive dyspnea | respiratory distress | shortness of breath | sob | worsening dyspnea | worsening sob
cough	24,018 (25.6)	14,600 (28.1)	534 (28.1)	1,659 (19.3)	1,225 (27.4)	chest congestion | chronic cough | cough productive | coughing | dry cough | increased cough | intermittent cough | mild cough | new cough | non productive cough | nonproductive cough | productive cough | worsening cough
abdominal pain	20,831 (22.2)	11,209 (21.5)	236 (12.4)	2,088 (24.2)	784 (17.6)	abd pain | abdominal cramping | abdominal discomfort | abdominal pain radiating | abdominal tenderness | acute abdominal pain | chronic abdominal pain | crampy abdominal pain | diffuse abdominal pain | epigastric abdominal pain | epigastric discomfort | epigastric pain | epigastric pain radiating | generalized abdominal pain | left flank pain | llq abdominal pain | llq pain | lower abdominal pain | lower quadrant pain | luq pain | mild abdominal pain | quadrant abdominal pain | rlq abdominal pain | rlq pain | ruq abdominal pain | ruq pain | severe abdominal pain | sided abdominal pain | suprapubic discomfort | suprapubic pain | suprapubic tenderness | upper quadrant pain
pain	20,300 (21.7)	9,491 (18.2)	492 (25.9)	1,447 (16.8)	479 (10.7)	aches | chronic pain | increased pain | increasing pain | pain radiating | painful swelling | pains | severe pain | sharp pain | worsening pain
nausea	20,036 (21.4)	10,881 (20.9)	252 (13.2)	1,773 (20.6)	648 (14.5)	intermittent nausea | mild nausea | n | nausea without | nausea without vomiting

© 2025 Pak TR et al. *JAMA Network Open*

eTable 2 (cont.)

	Patients with labeled admission notes, No. (%)					
	All	And with ≥1 positive culture[a]	And with MRSA in ≥1 culture[a]	And with MDRGN in ≥1 culture[a]	And with in-hospital mortality	All synonyms[b]
chills	19,923 (21.3)	11,725 (22.5)	362 (19.0)	2,094 (24.3)	343 (7.7)	feverish feeling | intermittent chills | rigor | rigors | shaking chills | shivering | subjective chills
fatigue	19,439 (20.8)	11,424 (22.0)	268 (14.1)	1,789 (20.8)	1,020 (22.8)	decreased activity | decreased activity level | decreased energy | decreased exercise | decreased exercise tolerance | difficulty moving | generalized fatigue | generalized weakness | increased fatigue | increased weakness | tiredness
vomiting	17,000 (18.1)	9,307 (17.9)	227 (11.9)	1,595 (18.5)	641 (14.4)	bilious emesis | bilious vomiting | coffee ground emesis | dry heaves | dry heaving | emesis | n v | n v d | nausea vomiting | nbnb emesis | nbnb vomiting | non bloody emesis | nonbloody emesis | v | v d | vomit | vomitus
altered mental status	16,010 (17.1)	10,033 (19.3)	341 (17.9)	2,024 (23.5)	1,304 (29.2)	cognitive impairment | confused | confusion | consciousness | decreased gcs | decreased mental status | decreased responsiveness | delirium | difficulty speaking | disorientation | encephalopathy | increased confusion | increased lethargy | increasing confusion | lethargic | lethargy | mental status change | mental status changes | non verbal | nonverbal | responsiveness | word finding difficulty
diarrhea	11,941 (12.7)	6,329 (12.2)	161 (8.5)	971 (11.3)	506 (11.3)	chronic diarrhea | d | intermittent diarrhea | loose bowel | loose bowel movements | loose stool | loose stools | n v d | non bloody diarrhea | nonbloody diarrhea | v d | watery diarrhea | watery stools
chest pain	9,503 (10.1)	5,230 (10.1)	221 (11.6)	632 (7.3)	459 (10.3)	chest discomfort | chest pain radiating | chest pressure | chest tightness | chest wall pain | cp | pleuritic chest pain | substernal chest pain
weakness	9,212 (9.8)	5,416 (10.4)	143 (7.5)	912 (10.6)	489 (11.0)	progressive weakness
swelling	8,817 (9.4)	3,351 (6.4)	321 (16.9)	343 (4.0)	188 (4.2)	edema | increased swelling | painful swelling | pitting edema | swollen
sputum changes	8,080 (8.6)	5,692 (10.9)	269 (14.1)	752 (8.7)	378 (8.5)	brown sputum | cough productive | green sputum | greenish sputum | increased secretions | increased sputum | increased sputum production | phlegm | phlegm production | productive cough | productive sputum | secretions | sputum production | tinged sputum | white phlegm | white sputum | whitish sputum | yellow green sputum | yellow phlegm |

© 2025 Pak TR et al. *JAMA Network Open*

eTable 2 (cont.)

	Patients with labeled admission notes, No. (%)					
	All	And with ≥1 positive culture[a]	And with MRSA in ≥1 culture[a]	And with MDRGN in ≥1 culture[a]	And with in-hospital mortality	All synonyms[b]
						yellow sputum | yellow sputum production | yellowish sputum
redness	7,928 (8.5)	2,645 (5.1)	301 (15.8)	289 (3.4)	86 (1.9)	bright red | erythema | erythematous | red | redness around
headache	7,335 (7.8)	3,932 (7.6)	111 (5.8)	489 (5.7)	148 (3.3)	frontal headache | ha | headaches | mild headache
poor appetite	7,275 (7.8)	4,274 (8.2)	105 (5.5)	670 (7.8)	367 (8.2)	anorexia | decreased appetite
malaise	7,207 (7.7)	4,308 (8.3)	126 (6.6)	665 (7.7)	263 (5.9)	general malaise | generalized malaise | unwell
back pain	5,974 (6.4)	3,582 (6.9)	151 (7.9)	530 (6.2)	215 (4.8)	back pain radiating | chronic back pain | lbp | low back pain | lower back pain
hypoxemia	4,921 (5.3)	3,111 (6.0)	169 (8.9)	552 (6.4)	716 (16.0)	decreased o2 | decreased oxygen | decreased oxygen saturation | desaturation | desaturations | hypoxemic | hypoxemic respiratory failure | hypoxia | hypoxic | hypoxic respiratory | hypoxic respiratory failure | increased o2 requirement | increased oxygen | increased oxygen requirement | new o2 requirement | o2 requirement | o2 sats | o2 saturation | oxygen saturation
hypotension	4,093 (4.4)	2,505 (4.8)	109 (5.7)	543 (6.3)	527 (11.8)	decreased blood pressure | hypotension requiring | hypotensive | low blood pressure | low bp | orthostatic hypotension | requiring pressors | septic shock
dizziness	4,009 (4.3)	2,110 (4.1)	39 (2.0)	280 (3.2)	141 (3.2)	
dysuria	3,964 (4.2)	3,087 (5.9)	24 (1.3)	629 (7.3)	59 (1.3)	painful urination
rhinorrhea	3,931 (4.2)	2,308 (4.4)	57 (3.0)	246 (2.9)	123 (2.8)	congestion | nasal congestion | runny nose | sinus congestion
lightheadedness	3,546 (3.8)	1,868 (3.6)	36 (1.9)	241 (2.8)	146 (3.3)	light headedness
fall	3,338 (3.6)	1,931 (3.7)	44 (2.3)	304 (3.5)	206 (4.6)	falling | fell | mechanical fall | multiple falls | recent fall | unwitnessed fall
urinary frequency	3,321 (3.5)	2,494 (4.8)	28 (1.5)	486 (5.6)	55 (1.2)	frequent urination | increased frequency | increased urinary frequency | increased urination | polyuria
diaphoresis	3,225 (3.4)	1,779 (3.4)	72 (3.8)	299 (3.5)	92 (2.1)	diaphoretic | sweating | sweats
leg swelling	3,100 (3.3)	1,296 (2.5)	50 (2.6)	155 (1.8)	160 (3.6)	bilateral le edema | bilateral leg swelling | le edema | le swelling | leg edema | lle swelling | lower extremity edema | lower extremity swelling | rle swelling
decreased po intake	3,091 (3.3)	1,842 (3.5)	51 (2.7)	315 (3.7)	261 (5.8)	decreased oral intake | decreased p o | decreased po | early satiety | poor oral intake | poor p o | poor po | poor po intake
constipation	3,082 (3.3)	1,748 (3.4)	34 (1.8)	296 (3.4)	134 (3.0)	chronic constipation

© 2025 Pak TR et al. *JAMA Network Open*

eTable 2 (cont.)

	Patients with labeled admission notes, No. (%)					
	All	And with ≥1 positive culture[a]	And with MRSA in ≥1 culture[a]	And with MDRGN in ≥1 culture[a]	And with in-hospital mortality	All synonyms[b]
shaking	3,080 (3.3)	1,857 (3.6)	38 (2.0)	350 (4.1)	64 (1.4)	jerking | shakes | shakiness | shaking chills
myalgia	2,974 (3.2)	1,653 (3.2)	58 (3.0)	148 (1.7)	66 (1.5)	diffuse myalgias | muscle aches | myalgias
tachycardia	2,951 (3.2)	1,797 (3.5)	90 (4.7)	411 (4.8)	248 (5.6)	complex tachycardia | increased heart rate | sinus tachycardia | tachycardia hr | tachycardic
sore throat	2,907 (3.1)	1,552 (3.0)	47 (2.5)	127 (1.5)	64 (1.4)	
leg pain	2,577 (2.8)	1,002 (1.9)	51 (2.7)	146 (1.7)	65 (1.5)	bilateral leg pain | left leg pain | lle pain | lower extremity pain | right leg pain | rle pain
flank pain	2,416 (2.6)	1,812 (3.5)	32 (1.7)	386 (4.5)	22 (0.5)	bilateral flank pain | flank pain radiating | l flank pain | r flank pain | right flank pain | sided flank pain
warmth	2,368 (2.5)	1,053 (2.0)	55 (2.9)	146 (1.7)	37 (0.8)	hot | warm
unresponsiveness	2,365 (2.5)	1,491 (2.9)	57 (3.0)	224 (2.6)	381 (8.5)	decreased loc | loc | obtundation | obtunded | unresponsive
weight loss	2,334 (2.5)	1,341 (2.6)	33 (1.7)	171 (2.0)	130 (2.9)	ftt | unintentional weight | unintentional weight loss
difficulty walking	2,304 (2.5)	1,227 (2.4)	32 (1.7)	168 (1.9)	98 (2.2)	ataxia | decreased mobility | difficulty ambulating | gait instability | unsteady gait
abdominal distension	2,298 (2.5)	1,146 (2.2)	16 (0.8)	192 (2.2)	174 (3.9)	abdominal bloating | abdominal distention | abdominal fullness | abdominal girth | bloating | distended abdomen | increased abdominal girth
bleeding	2,179 (2.3)	1,119 (2.2)	52 (2.7)	179 (2.1)	91 (2.0)	blood tinged | bloody | bright red blood | hemorrhage | hemorrhagic | red blood | red blood per
drainage	2,179 (2.3)	997 (1.9)	96 (5.0)	214 (2.5)	42 (0.9)	increased drainage | leakage
wound	2,121 (2.3)	765 (1.5)	73 (3.8)	107 (1.2)	64 (1.4)	sores | ulceration | ulcers | wounds
tenderness	1,976 (2.1)	843 (1.6)	64 (3.4)	107 (1.2)	33 (0.7)	tender
body aches	1,901 (2.0)	1,061 (2.0)	34 (1.8)	129 (1.5)	39 (0.9)	achiness | achy | body pain | diffuse body aches | generalized body aches
discomfort	1,867 (2.0)	999 (1.9)	50 (2.6)	165 (1.9)	57 (1.3)	
wheezing	1,843 (2.0)	1,076 (2.1)	29 (1.5)	98 (1.1)	83 (1.9)	wheezes
burning	1,803 (1.9)	1,162 (2.2)	23 (1.2)	234 (2.7)	23 (0.5)	burning sensation
somnolence	1,687 (1.8)	988 (1.9)	37 (1.9)	185 (2.1)	142 (3.2)	somnolent
tachypnea	1,634 (1.7)	1,081 (2.1)	52 (2.7)	209 (2.4)	243 (5.4)	increased respiratory rate | increased wob | labored breathing | tachypneic | wob
rash	1,605 (1.7)	662 (1.3)	39 (2.0)	79 (0.9)	36 (0.8)	rashes | skin changes | skin lesions
urine color change	1,600 (1.7)	1,228 (2.4)	35 (1.8)	367 (4.3)	41 (0.9)	cloudy urine | colored urine | dark urine
pleuritic pain	1,569 (1.7)	948 (1.8)	53 (2.8)	94 (1.1)	48 (1.1)	pleurisy | pleuritic chest pain | rib pain
cramping	1,490 (1.6)	658 (1.3)	15 (0.8)	79 (0.9)	36 (0.8)	cramps | crampy
night sweats	1,474 (1.6)	806 (1.5)	26 (1.4)	80 (0.9)	26 (0.6)	drenching night sweats
cold	1,440 (1.5)	829 (1.6)	16 (0.8)	117 (1.4)	48 (1.1)	

© 2025 Pak TR et al. *JAMA Network Open*

eTable 2 (cont.)

	Patients with labeled admission notes, No. (%)					
	All	And with ≥1 positive culture[a]	And with MRSA in ≥1 culture[a]	And with MDRGN in ≥1 culture[a]	And with in-hospital mortality	All synonyms[b]
dysphagia	1,431 (1.5)	781 (1.5)	34 (1.8)	98 (1.1)	106 (2.4)	choking | difficulty swallowing
hematuria	1,411 (1.5)	1,106 (2.1)	24 (1.3)	278 (3.2)	34 (0.8)	gross hematuria
incontinence	1,325 (1.4)	845 (1.6)	21 (1.1)	142 (1.6)	55 (1.2)	
agitation	1,318 (1.4)	773 (1.5)	30 (1.6)	148 (1.7)	80 (1.8)	agitated | combativeness
pressure	1,277 (1.4)	693 (1.3)	26 (1.4)	115 (1.3)	47 (1.1)	pressures
neck pain	1,269 (1.4)	624 (1.2)	42 (2.2)	72 (0.8)	26 (0.6)	
purulence	1,264 (1.3)	574 (1.1)	104 (5.5)	94 (1.1)	10 (0.2)	purulent discharge | purulent drainage | pus
urinary tract infection	1,202 (1.3)	994 (1.9)	24 (1.3)	323 (3.7)	42 (0.9)	recurrent uti | recurrent utis | uti | utis
bloody stool	1,193 (1.3)	482 (0.9)	9 (0.5)	72 (0.8)	81 (1.8)	blood per rectum | bloody diarrhea | bloody stools | brbpr | gi bleed | gib | hematochezia | melena | rectal bleeding
foot pain	1,166 (1.2)	386 (0.7)	37 (1.9)	33 (0.4)	18 (0.4)	left foot pain | right foot pain
pruritus	1,124 (1.2)	461 (0.9)	21 (1.1)	58 (0.7)	16 (0.4)	itchiness | itching | itchy | pruritic | pruritis
palpitations	1,093 (1.2)	546 (1.0)	3 (0.2)	81 (0.9)	43 (1.0)	palpitation
trauma	1,088 (1.2)	543 (1.0)	20 (1.1)	76 (0.9)	70 (1.6)	fracture | fractures | head injury | head strike | head trauma | headstrike | laceration | rib fractures | traumatic
syncope	1,070 (1.1)	524 (1.0)	9 (0.5)	65 (0.8)	56 (1.3)	syncopal | syncopal episode | syncopal episodes
numbness	1,065 (1.1)	442 (0.8)	18 (0.9)	48 (0.6)	18 (0.4)	decreased sensation
seizure	1,028 (1.1)	598 (1.1)	22 (1.2)	113 (1.3)	71 (1.6)	gtc | seizure activity | seizure disorder | seizure like | seizure like activity | seizures | tonic clonic
discharge	995 (1.1)	446 (0.9)	34 (1.8)	72 (0.8)	10 (0.2)	
knee pain	990 (1.1)	476 (0.9)	48 (2.5)	36 (0.4)	18 (0.4)	bilateral knee pain | left knee pain | right knee pain
urinary incontinence	959 (1.0)	663 (1.3)	14 (0.7)	113 (1.3)	20 (0.4)	
hip pain	955 (1.0)	541 (1.0)	32 (1.7)	78 (0.9)	28 (0.6)	left hip pain | right hip pain
orthopnea	944 (1.0)	505 (1.0)	12 (0.6)	40 (0.5)	60 (1.3)	
malodorous urine	937 (1.0)	800 (1.5)	17 (0.9)	241 (2.8)	21 (0.5)	foul smelling urine | smelling urine
shoulder pain	928 (1.0)	510 (1.0)	33 (1.7)	60 (0.7)	25 (0.6)	left shoulder pain | right shoulder pain
malodorous	878 (0.9)	493 (0.9)	28 (1.5)	106 (1.2)	18 (0.4)	foul odor | foul smelling | odor | smelling
urinary urgency	878 (0.9)	685 (1.3)	6 (0.3)	134 (1.6)	9 (0.2)	
cellulitis	837 (0.9)	238 (0.5)	29 (1.5)	35 (0.4)	17 (0.4)	
leukocytosis	818 (0.9)	524 (1.0)	28 (1.5)	132 (1.5)	65 (1.5)	elevated wbc | elevated wbc count | increased wbc | neutrophilia
hemoptysis	817 (0.9)	618 (1.2)	38 (2.0)	65 (0.8)	64 (1.4)	blood tinged sputum

© 2025 Pak TR et al. *JAMA Network Open*

eTable 2 (cont.)

	Patients with labeled admission notes, No. (%)					
	All	And with ≥1 positive culture[a]	And with MRSA in ≥1 culture[a]	And with MDRGN in ≥1 culture[a]	And with in-hospital mortality	All synonyms[b]
urinary retention	798 (0.9)	572 (1.1)	17 (0.9)	179 (2.1)	26 (0.6)	
leg weakness	790 (0.8)	471 (0.9)	15 (0.8)	81 (0.9)	45 (1.0)	le weakness | lower extremity weakness
anxiety	786 (0.8)	421 (0.8)	19 (1.0)	69 (0.8)	52 (1.2)	
urgency	762 (0.8)	561 (1.1)	2 (0.1)	107 (1.2)	7 (0.2)	
dark stools	723 (0.8)	371 (0.7)	7 (0.4)	42 (0.5)	43 (1.0)	black stool | black stools | gi bleeding | tarry stools
oliguria	703 (0.8)	493 (0.9)	6 (0.3)	132 (1.5)	46 (1.0)	decreased uop | decreased urinary output | decreased urine | decreased urine output
dysarthria	679 (0.7)	405 (0.8)	11 (0.6)	64 (0.7)	58 (1.3)	slurred speech
hallucinations	676 (0.7)	382 (0.7)	7 (0.4)	64 (0.7)	50 (1.1)	auditory hallucinations | visual hallucinations
sore	672 (0.7)	343 (0.7)	11 (0.6)	55 (0.6)	16 (0.4)	soreness
weight gain	639 (0.7)	298 (0.6)	9 (0.5)	28 (0.3)	28 (0.6)	
alcohol	621 (0.7)	321 (0.6)	9 (0.5)	45 (0.5)	39 (0.9)	alcohol abuse | alcohol intoxication | alcohol use | alcohol use disorder | alcohol withdrawal | alcoholic | aud | drinking | etoh | etoh use
infection	618 (0.7)	264 (0.5)	28 (1.5)	48 (0.6)	21 (0.5)	infected | infections | tract infection
arm pain	617 (0.7)	269 (0.5)	16 (0.8)	26 (0.3)	13 (0.3)	
tremor	607 (0.6)	352 (0.7)	11 (0.6)	67 (0.8)	16 (0.4)	tremors | tremulousness
thirst	600 (0.6)	367 (0.7)	15 (0.8)	60 (0.7)	33 (0.7)	dry mouth | increased thirst | polydipsia
dehydration	597 (0.6)	356 (0.7)	9 (0.5)	57 (0.7)	39 (0.9)	
arthralgia	594 (0.6)	306 (0.6)	12 (0.6)	21 (0.2)	6 (0.1)	arthralgias | joint pain
memory loss	580 (0.6)	333 (0.6)	5 (0.3)	48 (0.6)	33 (0.7)	dementia | forgetfulness
jaundice	548 (0.6)	271 (0.5)	5 (0.3)	48 (0.6)	48 (1.1)	
difficulty urinating	546 (0.6)	380 (0.7)	8 (0.4)	72 (0.8)	13 (0.3)	neurogenic bladder | urinary hesitancy
hematemesis	537 (0.6)	230 (0.4)	4 (0.2)	35 (0.4)	33 (0.7)	bloody emesis | ground emesis | vomiting blood
abscess	518 (0.6)	281 (0.5)	71 (3.7)	23 (0.3)	2 (0.0)	abscesses
focal weakness	511 (0.5)	309 (0.6)	11 (0.6)	40 (0.5)	29 (0.6)	arm weakness | hemiparesis | l sided weakness | left sided weakness | right sided weakness | sided weakness
hypertension	506 (0.5)	291 (0.6)	12 (0.6)	64 (0.7)	31 (0.7)	htn | hypertensive
vision changes	498 (0.5)	220 (0.4)	8 (0.4)	15 (0.2)	10 (0.2)	blurred vision | blurry vision | visual changes | visual disturbances
difficulty sleeping	498 (0.5)	256 (0.5)	6 (0.3)	22 (0.3)	31 (0.7)	
tingling	496 (0.5)	213 (0.4)	10 (0.5)	23 (0.3)	4 (0.1)	
respiratory failure	478 (0.5)	338 (0.6)	19 (1.0)	70 (0.8)	106 (2.4)	acute respiratory failure | bipap | failure requiring intubation | hypercarbic respiratory failure | hypoxemic respiratory failure | intubated | intubation

© 2025 Pak TR et al. *JAMA Network Open*

eTable 2 (cont.)

	Patients with labeled admission notes, No. (%)					
	All	And with ≥1 positive culture[a]	And with MRSA in ≥1 culture[a]	And with MDRGN in ≥1 culture[a]	And with in-hospital mortality	All synonyms[b]
						| nrb | requiring intubation | respiratory failure requiring
glucose	475 (0.5)	258 (0.5)	8 (0.4)	34 (0.4)	32 (0.7)	blood glucose | blood sugar | blood sugars | sugars
hemoglobin	462 (0.5)	247 (0.5)	8 (0.4)	45 (0.5)	42 (0.9)	anemia | h h | hct | hgb
aspiration	451 (0.5)	303 (0.6)	30 (1.6)	79 (0.9)	62 (1.4)	aspiration event | aspiration pna
ankle pain	450 (0.5)	187 (0.4)	14 (0.7)	14 (0.2)	4 (0.1)	
groin pain	430 (0.5)	235 (0.5)	12 (0.6)	42 (0.5)	14 (0.3)	
depression	418 (0.4)	218 (0.4)	12 (0.6)	34 (0.4)	12 (0.3)	depressed | depressed mood
gas	415 (0.4)	193 (0.4)	5 (0.3)	36 (0.4)	18 (0.4)	
cardiac arrest	406 (0.4)	268 (0.5)	7 (0.4)	23 (0.3)	133 (3.0)	asystole | pea | pea arrest | vf | vf arrest | vt
mucus	401 (0.4)	265 (0.5)	20 (1.1)	53 (0.6)	19 (0.4)	
throbbing	396 (0.4)	164 (0.3)	10 (0.5)	16 (0.2)	3 (0.1)	
hand pain	394 (0.4)	134 (0.3)	17 (0.9)	6 (0.1)	0 (0.0)	
distension	379 (0.4)	199 (0.4)	2 (0.1)	41 (0.5)	27 (0.6)	distended | distention
heartburn	370 (0.4)	186 (0.4)	4 (0.2)	27 (0.3)	16 (0.4)	gerd | reflux
unsteadiness	370 (0.4)	192 (0.4)	3 (0.2)	24 (0.3)	24 (0.5)	
blister	368 (0.4)	120 (0.2)	7 (0.4)	12 (0.1)	4 (0.1)	blisters
pneumonia	365 (0.4)	206 (0.4)	20 (1.1)	52 (0.6)	46 (1.0)	aspiration pna | aspiration pneumonia | pna
hyperglycemia	364 (0.4)	216 (0.4)	5 (0.3)	39 (0.5)	12 (0.3)	
thigh pain	351 (0.4)	173 (0.3)	10 (0.5)	16 (0.2)	6 (0.1)	
pallor	347 (0.4)	183 (0.4)	10 (0.5)	30 (0.3)	31 (0.7)	pale
smell	346 (0.4)	184 (0.4)	1 (0.1)	38 (0.4)	11 (0.2)	
hypoglycemia	329 (0.4)	190 (0.4)	6 (0.3)	24 (0.3)	43 (1.0)	
bruising	329 (0.4)	144 (0.3)	8 (0.4)	20 (0.2)	26 (0.6)	ecchymosis
foot swelling	327 (0.3)	90 (0.2)	11 (0.6)	11 (0.1)	1 (0.0)	
ear pain	317 (0.3)	102 (0.2)	3 (0.2)	4 (0.0)	3 (0.1)	
nocturnal dyspnea	316 (0.3)	153 (0.3)	2 (0.1)	14 (0.2)	11 (0.2)	paroxysmal nocturnal dyspnea | pnd
clots	316 (0.3)	205 (0.4)	8 (0.4)	40 (0.5)	15 (0.3)	emboli | embolism | thrombosis | thrombus
bradycardia	314 (0.3)	188 (0.4)	6 (0.3)	33 (0.4)	40 (0.9)	
photophobia	309 (0.3)	125 (0.2)	5 (0.3)	9 (0.1)	2 (0.0)	
pelvic pain	304 (0.3)	194 (0.4)	0 (0.0)	35 (0.4)	3 (0.1)	
facial swelling	303 (0.3)	104 (0.2)	16 (0.8)	12 (0.1)	7 (0.2)	
hypothermia	297 (0.3)	168 (0.3)	2 (0.1)	44 (0.5)	34 (0.8)	
acute kidney injury	289 (0.3)	169 (0.3)	7 (0.4)	40 (0.5)	23 (0.5)	acute renal failure | aki
neutropenia	289 (0.3)	156 (0.3)	1 (0.1)	21 (0.2)	18 (0.4)	febrile neutropenia
tightness	282 (0.3)	131 (0.3)	2 (0.1)	17 (0.2)	6 (0.1)	

© 2025 Pak TR et al. *JAMA Network Open*

eTable 2 (cont.)

	Patients with labeled admission notes, No. (%)					
	All	And with ≥1 positive culture[a]	And with MRSA in ≥1 culture[a]	And with MDRGN in ≥1 culture[a]	And with in-hospital mortality	All synonyms[b]
odynophagia	280 (0.3)	126 (0.2)	6 (0.3)	12 (0.1)	7 (0.2)	painful swallowing
facial droop	279 (0.3)	176 (0.3)	5 (0.3)	37 (0.4)	18 (0.4)	sided facial droop
stiffness	272 (0.3)	127 (0.2)	3 (0.2)	13 (0.2)	3 (0.1)	rigidity
sepsis	271 (0.3)	193 (0.4)	11 (0.6)	54 (0.6)	37 (0.8)	septic | septic shock
rectal pain	266 (0.3)	137 (0.3)	6 (0.3)	31 (0.4)	5 (0.1)	
upper respiratory tract infection	251 (0.3)	159 (0.3)	2 (0.1)	15 (0.2)	9 (0.2)	uri | uri symptoms
presyncope	249 (0.3)	121 (0.2)	2 (0.1)	13 (0.2)	13 (0.3)	near syncope
throat pain	249 (0.3)	106 (0.2)	0 (0.0)	10 (0.1)	8 (0.2)	
lactate	248 (0.3)	152 (0.3)	2 (0.1)	34 (0.4)	31 (0.7)	elevated lactate | lactate elevated | lactate elevation | lactic | lactic acid | lactic acidosis
arm swelling	239 (0.3)	89 (0.2)	10 (0.5)	8 (0.1)	4 (0.1)	
discoloration	224 (0.2)	81 (0.2)	6 (0.3)	8 (0.1)	7 (0.2)	
buttock pain	222 (0.2)	141 (0.3)	13 (0.7)	23 (0.3)	5 (0.1)	
neck stiffness	221 (0.2)	89 (0.2)	2 (0.1)	8 (0.1)	2 (0.0)	
foot wound	221 (0.2)	68 (0.1)	5 (0.3)	8 (0.1)	2 (0.0)	foot ulcer
calf pain	220 (0.2)	89 (0.2)	8 (0.4)	10 (0.1)	6 (0.1)	
suicidal	218 (0.2)	86 (0.2)	6 (0.3)	8 (0.1)	1 (0.0)	si | suicidal ideation
ankle swelling	218 (0.2)	88 (0.2)	2 (0.1)	12 (0.1)	3 (0.1)	ankle edema
jaw pain	217 (0.2)	83 (0.2)	7 (0.4)	11 (0.1)	6 (0.1)	
elbow pain	216 (0.2)	92 (0.2)	8 (0.4)	5 (0.1)	4 (0.1)	
influenza	216 (0.2)	118 (0.2)	2 (0.1)	11 (0.1)	14 (0.3)	flu
hypercarbia	215 (0.2)	148 (0.3)	13 (0.7)	30 (0.3)	32 (0.7)	hypercarbic | hypercarbic respiratory | hypercarbic respiratory failure
clamminess	213 (0.2)	113 (0.2)	5 (0.3)	22 (0.3)	16 (0.4)	clammy | cool
atrial fibrillation	211 (0.2)	135 (0.3)	7 (0.4)	20 (0.2)	37 (0.8)	af | afib | rvr
hyponatremia	210 (0.2)	115 (0.2)	2 (0.1)	18 (0.2)	15 (0.3)	
weeping	206 (0.2)	67 (0.1)	5 (0.3)	15 (0.2)	8 (0.2)	
heaviness	203 (0.2)	111 (0.2)	1 (0.1)	14 (0.2)	5 (0.1)	
hoarseness	202 (0.2)	104 (0.2)	2 (0.1)	5 (0.1)	5 (0.1)	
insomnia	199 (0.2)	105 (0.2)	1 (0.1)	9 (0.1)	10 (0.2)	
fecal incontinence	197 (0.2)	116 (0.2)	1 (0.1)	21 (0.2)	12 (0.3)	
aphasia	195 (0.2)	120 (0.2)	1 (0.1)	20 (0.2)	17 (0.4)	
pink	195 (0.2)	123 (0.2)	2 (0.1)	14 (0.2)	10 (0.2)	
withdrawal	193 (0.2)	80 (0.2)	9 (0.5)	6 (0.1)	4 (0.1)	alcohol withdrawal
mucous	192 (0.2)	121 (0.2)	8 (0.4)	30 (0.3)	11 (0.2)	

© 2025 Pak TR et al. *JAMA Network Open*

eTable 2 (cont.)

	Patients with labeled admission notes, No. (%)					
	All	And with ≥1 positive culture[a]	And with MRSA in ≥1 culture[a]	And with MDRGN in ≥1 culture[a]	And with in-hospital mortality	All synonyms[b]
neck swelling	188 (0.2)	69 (0.1)	10 (0.5)	5 (0.1)	2 (0.0)	
gurgling	188 (0.2)	122 (0.2)	8 (0.4)	20 (0.2)	25 (0.6)	
sneezing	186 (0.2)	104 (0.2)	2 (0.1)	7 (0.1)	4 (0.1)	
urinary symptoms	185 (0.2)	143 (0.3)	1 (0.1)	31 (0.4)	3 (0.1)	
ostomy output	183 (0.2)	121 (0.2)	4 (0.2)	28 (0.3)	5 (0.1)	
drug use	180 (0.2)	66 (0.1)	7 (0.4)	5 (0.1)	5 (0.1)	cocaine | cocaine use | heroin | heroin use | iv drug | iv drug use | ivdu | opiate | opioid | opioid use | overdose | polysubstance abuse | polysubstance use | psud
twitching	180 (0.2)	106 (0.2)	3 (0.2)	24 (0.3)	4 (0.1)	
facial pain	177 (0.2)	62 (0.1)	5 (0.3)	6 (0.1)	3 (0.1)	
lesions	171 (0.2)	74 (0.1)	10 (0.5)	6 (0.1)	5 (0.1)	
epistaxis	170 (0.2)	83 (0.2)	4 (0.2)	9 (0.1)	14 (0.3)	
intoxication	167 (0.2)	72 (0.1)	3 (0.2)	12 (0.1)	4 (0.1)	impaired
irritation	164 (0.2)	90 (0.2)	3 (0.2)	19 (0.2)	0 (0.0)	
ascites	161 (0.2)	70 (0.1)	0 (0.0)	6 (0.1)	20 (0.4)	
palpation	160 (0.2)	52 (0.1)	3 (0.2)	8 (0.1)	3 (0.1)	
necrosis	158 (0.2)	56 (0.1)	4 (0.2)	10 (0.1)	4 (0.1)	necrotic | necrotizing
restlessness	155 (0.2)	88 (0.2)	2 (0.1)	16 (0.2)	19 (0.4)	
apnea	146 (0.2)	96 (0.2)	2 (0.1)	14 (0.2)	26 (0.6)	apneic
diabetes	143 (0.2)	56 (0.1)	1 (0.1)	3 (0.0)	5 (0.1)	diabetes mellitus | diabetes mellitus type | diabetic | dka | dm | dm2 | iddm | t2dm
hyperkalemia	142 (0.2)	79 (0.2)	1 (0.1)	23 (0.3)	12 (0.3)	
vertigo	141 (0.2)	76 (0.1)	0 (0.0)	6 (0.1)	3 (0.1)	
cyanosis	139 (0.1)	87 (0.2)	2 (0.1)	13 (0.2)	18 (0.4)	
lump	139 (0.1)	68 (0.1)	7 (0.4)	7 (0.1)	3 (0.1)	
induration	134 (0.1)	54 (0.1)	7 (0.4)	7 (0.1)	1 (0.0)	
vaginal bleeding	129 (0.1)	77 (0.1)	0 (0.0)	10 (0.1)	4 (0.1)	
testicular pain	125 (0.1)	75 (0.1)	2 (0.1)	15 (0.2)	0 (0.0)	
extremity pain	122 (0.1)	45 (0.1)	1 (0.1)	3 (0.0)	2 (0.0)	peripheral neuropathy
nocturia	121 (0.1)	85 (0.2)	2 (0.1)	18 (0.2)	0 (0.0)	
scrotal pain	117 (0.1)	64 (0.1)	2 (0.1)	8 (0.1)	3 (0.1)	
crackles	116 (0.1)	72 (0.1)	5 (0.3)	15 (0.2)	16 (0.4)	rales
scrotal swelling	113 (0.1)	64 (0.1)	1 (0.1)	12 (0.1)	1 (0.0)	
wound drainage	112 (0.1)	48 (0.1)	7 (0.4)	7 (0.1)	1 (0.0)	
distress	111 (0.1)	65 (0.1)	1 (0.1)	11 (0.1)	9 (0.2)	

© 2025 Pak TR et al. *JAMA Network Open*

eTable 2 (cont.)

	Patients with labeled admission notes, No. (%)					
	All	And with ≥1 positive culture[a]	And with MRSA in ≥1 culture[a]	And with MDRGN in ≥1 culture[a]	And with in-hospital mortality	All synonyms[b]
hematoma	108 (0.1)	46 (0.1)	0 (0.0)	10 (0.1)	6 (0.1)	
pyuria	107 (0.1)	91 (0.2)	4 (0.2)	29 (0.3)	4 (0.1)	
paranoia	107 (0.1)	54 (0.1)	2 (0.1)	13 (0.2)	3 (0.1)	
pinpoint pupils	106 (0.1)	60 (0.1)	0 (0.0)	4 (0.0)	7 (0.2)	
hiccups	106 (0.1)	54 (0.1)	1 (0.1)	10 (0.1)	5 (0.1)	
paresthesias	103 (0.1)	45 (0.1)	2 (0.1)	8 (0.1)	2 (0.0)	
flushing	103 (0.1)	54 (0.1)	1 (0.1)	12 (0.1)	1 (0.0)	
neuropathy	102 (0.1)	35 (0.1)	2 (0.1)	4 (0.0)	2 (0.0)	
extremity swelling	101 (0.1)	57 (0.1)	6 (0.3)	9 (0.1)	6 (0.1)	peripheral edema
orthostatic	99 (0.1)	57 (0.1)	4 (0.2)	9 (0.1)	5 (0.1)	
volume overload	98 (0.1)	56 (0.1)	5 (0.3)	12 (0.1)	8 (0.2)	
sensitivity	97 (0.1)	44 (0.1)	0 (0.0)	1 (0.0)	2 (0.0)	
hesitancy	96 (0.1)	74 (0.1)	0 (0.0)	16 (0.2)	1 (0.0)	
post nasal drip	92 (0.1)	58 (0.1)	0 (0.0)	9 (0.1)	0 (0.0)	
pleural effusion	91 (0.1)	47 (0.1)	1 (0.1)	10 (0.1)	12 (0.3)	bilateral pleural effusions | pleural effusions
retention	91 (0.1)	57 (0.1)	3 (0.2)	12 (0.1)	3 (0.1)	
thrombocytopenia	90 (0.1)	41 (0.1)	1 (0.1)	7 (0.1)	7 (0.2)	
renal failure	89 (0.1)	52 (0.1)	4 (0.2)	10 (0.1)	11 (0.2)	dialysis
gaze deviation	89 (0.1)	58 (0.1)	2 (0.1)	8 (0.1)	8 (0.2)	
respiratory symptoms	87 (0.1)	55 (0.1)	3 (0.2)	10 (0.1)	8 (0.2)	
extremity weakness	86 (0.1)	49 (0.1)	3 (0.2)	7 (0.1)	9 (0.2)	
streaks	86 (0.1)	54 (0.1)	6 (0.3)	10 (0.1)	3 (0.1)	
metabolic acidosis	85 (0.1)	43 (0.1)	0 (0.0)	4 (0.0)	8 (0.2)	anion gap metabolic | dka | gap metabolic acidosis
regurgitation	84 (0.1)	48 (0.1)	1 (0.1)	9 (0.1)	1 (0.0)	
cerebrovascular accident	83 (0.1)	60 (0.1)	3 (0.2)	13 (0.2)	4 (0.1)	cva | stroke | tia
vaginal discharge	80 (0.1)	54 (0.1)	2 (0.1)	5 (0.1)	1 (0.0)	
transaminitis	79 (0.1)	34 (0.1)	0 (0.0)	4 (0.0)	8 (0.2)	elevated alt | elevated ast | elevated lfts | elevated liver enzymes
joint pain	79 (0.1)	39 (0.1)	0 (0.0)	7 (0.1)	2 (0.0)	joint pains
obstruction	77 (0.1)	53 (0.1)	0 (0.0)	24 (0.3)	5 (0.1)	
gangrene	77 (0.1)	21 (0.0)	1 (0.1)	7 (0.1)	6 (0.1)	
hypernatremia	76 (0.1)	51 (0.1)	1 (0.1)	15 (0.2)	10 (0.2)	
wound infection	75 (0.1)	31 (0.1)	1 (0.1)	10 (0.1)	3 (0.1)	

© 2025 Pak TR et al. *JAMA Network Open*

eTable 2 (cont.)

	Patients with labeled admission notes, No. (%)					
	All	And with ≥1 positive culture[a]	And with MRSA in ≥1 culture[a]	And with MDRGN in ≥1 culture[a]	And with in-hospital mortality	All synonyms[b]
agonal breathing	75 (0.1)	49 (0.1)	2 (0.1)	4 (0.0)	19 (0.4)	
wbc count	74 (0.1)	46 (0.1)	3 (0.2)	10 (0.1)	5 (0.1)	wbc | wbcs | white blood cell | white count
bone pain	74 (0.1)	39 (0.1)	0 (0.0)	4 (0.0)	3 (0.1)	
mucositis	69 (0.1)	22 (0.0)	0 (0.0)	0 (0.0)	3 (0.1)	
hydronephrosis	65 (0.1)	54 (0.1)	1 (0.1)	17 (0.2)	1 (0.0)	
nodule	65 (0.1)	31 (0.1)	5 (0.3)	3 (0.0)	1 (0.0)	nodules
pulmonary edema	61 (0.1)	35 (0.1)	2 (0.1)	7 (0.1)	7 (0.2)	
sacral wound	61 (0.1)	46 (0.1)	0 (0.0)	13 (0.2)	3 (0.1)	decubitus ulcer | sacral decubitus
dehiscence	61 (0.1)	24 (0.0)	2 (0.1)	4 (0.0)	1 (0.0)	
anosmia	58 (0.1)	26 (0.0)	1 (0.1)	0 (0.0)	0 (0.0)	
lymphadenopathy	56 (0.1)	26 (0.0)	3 (0.2)	3 (0.0)	0 (0.0)	
urinary output	55 (0.1)	41 (0.1)	1 (0.1)	10 (0.1)	2 (0.0)	uop
hypokalemia	54 (0.1)	27 (0.1)	2 (0.1)	6 (0.1)	4 (0.1)	
hernia	54 (0.1)	25 (0.0)	0 (0.0)	4 (0.0)	2 (0.0)	
osteomyelitis	53 (0.1)	22 (0.0)	0 (0.0)	7 (0.1)	2 (0.0)	
impairment	52 (0.1)	25 (0.0)	1 (0.1)	3 (0.0)	2 (0.0)	
delusions	52 (0.1)	34 (0.1)	1 (0.1)	5 (0.1)	2 (0.0)	
stridor	51 (0.1)	28 (0.1)	1 (0.1)	3 (0.0)	0 (0.0)	
fluid collection	50 (0.1)	24 (0.0)	2 (0.1)	3 (0.0)	2 (0.0)	
pyelonephritis	47 (0.1)	39 (0.1)	1 (0.1)	11 (0.1)	1 (0.0)	
diplopia	47 (0.1)	22 (0.0)	0 (0.0)	5 (0.1)	1 (0.0)	
bite	44 (0.0)	11 (0.0)	1 (0.1)	0 (0.0)	0 (0.0)	
stress	44 (0.0)	25 (0.0)	2 (0.1)	1 (0.0)	2 (0.0)	
stone	43 (0.0)	38 (0.1)	1 (0.1)	12 (0.1)	1 (0.0)	stones
hearing loss	41 (0.0)	16 (0.0)	1 (0.1)	2 (0.0)	2 (0.0)	
instability	41 (0.0)	21 (0.0)	2 (0.1)	1 (0.0)	1 (0.0)	
paralysis	41 (0.0)	22 (0.0)	3 (0.2)	4 (0.0)	1 (0.0)	
phonophobia	41 (0.0)	15 (0.0)	0 (0.0)	1 (0.0)	0 (0.0)	
liver function tests	39 (0.0)	23 (0.0)	0 (0.0)	5 (0.1)	2 (0.0)	alk | alk phos | alt | ast | lfts | liver enzymes
anasarca	38 (0.0)	19 (0.0)	1 (0.1)	5 (0.1)	4 (0.1)	
pancytopenia	35 (0.0)	18 (0.0)	0 (0.0)	3 (0.0)	2 (0.0)	
tinnitus	35 (0.0)	13 (0.0)	1 (0.1)	2 (0.0)	1 (0.0)	
hyperbilirubinemia	35 (0.0)	18 (0.0)	1 (0.1)	2 (0.0)	5 (0.1)	
asthma	34 (0.0)	23 (0.0)	2 (0.1)	3 (0.0)	1 (0.0)	
heart failure	31 (0.0)	19 (0.0)	2 (0.1)	6 (0.1)	3 (0.1)	chf | hfpef | hfref
urinalysis	26 (0.0)	20 (0.0)	0 (0.0)	5 (0.1)	1 (0.0)	nitrites | positive ua | positive urine | ua

© 2025 Pak TR et al. *JAMA Network Open*

eTable 2 (cont.)

	Patients with labeled admission notes, No. (%)					
	All	And with ≥1 positive culture[a]	And with MRSA in ≥1 culture[a]	And with MDRGN in ≥1 culture[a]	And with in-hospital mortality	All synonyms[b]
arthritis	25 (0.0)	9 (0.0)	0 (0.0)	2 (0.0)	1 (0.0)	
leukopenia	25 (0.0)	15 (0.0)	1 (0.1)	2 (0.0)	2 (0.0)	
hypercalcemia	25 (0.0)	12 (0.0)	0 (0.0)	2 (0.0)	4 (0.1)	
decreased breath sounds	24 (0.0)	13 (0.0)	0 (0.0)	3 (0.0)	5 (0.1)	decreased breath
labs	24 (0.0)	16 (0.0)	1 (0.1)	4 (0.0)	2 (0.0)	
infiltrates	24 (0.0)	11 (0.0)	1 (0.1)	1 (0.0)	2 (0.0)	
colitis	23 (0.0)	12 (0.0)	1 (0.1)	3 (0.0)	1 (0.0)	
atelectasis	23 (0.0)	11 (0.0)	1 (0.1)	6 (0.1)	5 (0.1)	
gout	23 (0.0)	9 (0.0)	0 (0.0)	1 (0.0)	1 (0.0)	
ischemia	23 (0.0)	10 (0.0)	0 (0.0)	2 (0.0)	4 (0.1)	
hemorrhoids	23 (0.0)	12 (0.0)	0 (0.0)	1 (0.0)	1 (0.0)	
ileus	23 (0.0)	18 (0.0)	1 (0.1)	9 (0.1)	4 (0.1)	
rhabdomyolysis	23 (0.0)	13 (0.0)	0 (0.0)	2 (0.0)	1 (0.0)	
pulmonary embolism	22 (0.0)	11 (0.0)	0 (0.0)	2 (0.0)	2 (0.0)	pe
troponinemia	21 (0.0)	14 (0.0)	1 (0.1)	3 (0.0)	1 (0.0)	elevated troponin | nstemi | troponin elevation
bilirubin	21 (0.0)	10 (0.0)	0 (0.0)	1 (0.0)	2 (0.0)	elevated bilirubin
consolidation	21 (0.0)	11 (0.0)	1 (0.1)	3 (0.0)	0 (0.0)	
blood culture	20 (0.0)	12 (0.0)	1 (0.1)	2 (0.0)	1 (0.0)	blood cultures | gnr bacteremia | mrsa bacteremia | mssa bacteremia | positive blood | positive blood cultures
deep vein thrombosis	20 (0.0)	9 (0.0)	0 (0.0)	3 (0.0)	2 (0.0)	dvt | dvts
urosepsis	20 (0.0)	16 (0.0)	0 (0.0)	9 (0.1)	0 (0.0)	
kidney injury	19 (0.0)	8 (0.0)	2 (0.1)	2 (0.0)	5 (0.1)	
abuse	19 (0.0)	13 (0.0)	0 (0.0)	5 (0.1)	1 (0.0)	
potassium	19 (0.0)	8 (0.0)	0 (0.0)	2 (0.0)	1 (0.0)	
cancer	18 (0.0)	10 (0.0)	0 (0.0)	1 (0.0)	3 (0.1)	cancer s p | carcinoma | metastases | metastatic | metastatic disease
perforation	18 (0.0)	7 (0.0)	0 (0.0)	0 (0.0)	3 (0.1)	perforated
mrsa	17 (0.0)	6 (0.0)	2 (0.1)	0 (0.0)	0 (0.0)	
lymphedema	17 (0.0)	7 (0.0)	0 (0.0)	0 (0.0)	0 (0.0)	
cyst	17 (0.0)	12 (0.0)	3 (0.2)	1 (0.0)	0 (0.0)	
hepatic encephalopathy	16 (0.0)	10 (0.0)	0 (0.0)	0 (0.0)	2 (0.0)	

eTable 2 (cont.)

	Patients with labeled admission notes, No. (%)					
	All	And with ≥1 positive culture[a]	And with MRSA in ≥1 culture[a]	And with MDRGN in ≥1 culture[a]	And with in-hospital mortality	All synonyms[b]
international normalized ratio	16 (0.0)	8 (0.0)	1 (0.1)	2 (0.0)	2 (0.0)	elevated inr | inr
venous stasis	16 (0.0)	4 (0.0)	0 (0.0)	1 (0.0)	0 (0.0)	
copd exacerbation	15 (0.0)	9 (0.0)	2 (0.1)	1 (0.0)	3 (0.1)	
sodium	15 (0.0)	10 (0.0)	0 (0.0)	1 (0.0)	1 (0.0)	na
chest x ray	14 (0.0)	8 (0.0)	1 (0.1)	1 (0.0)	1 (0.0)	cxr showed
covid	14 (0.0)	8 (0.0)	1 (0.1)	2 (0.0)	0 (0.0)	covid 19
viral symptoms	14 (0.0)	7 (0.0)	1 (0.1)	0 (0.0)	0 (0.0)	viral
hyperthermia	14 (0.0)	10 (0.0)	0 (0.0)	3 (0.0)	0 (0.0)	
pancreatitis	13 (0.0)	3 (0.0)	0 (0.0)	1 (0.0)	0 (0.0)	
nystagmus	13 (0.0)	8 (0.0)	0 (0.0)	1 (0.0)	1 (0.0)	
psychosis	12 (0.0)	5 (0.0)	0 (0.0)	0 (0.0)	1 (0.0)	
pneumonitis	12 (0.0)	6 (0.0)	0 (0.0)	1 (0.0)	0 (0.0)	
pneumoperitoneum	12 (0.0)	7 (0.0)	0 (0.0)	1 (0.0)	5 (0.1)	
hypercapnia	12 (0.0)	5 (0.0)	0 (0.0)	1 (0.0)	2 (0.0)	
c reactive protein	11 (0.0)	5 (0.0)	1 (0.1)	2 (0.0)	0 (0.0)	crp | elevated crp
respiratory acidosis	11 (0.0)	4 (0.0)	0 (0.0)	0 (0.0)	1 (0.0)	
cholangitis	11 (0.0)	5 (0.0)	0 (0.0)	1 (0.0)	0 (0.0)	
clostridium difficile	10 (0.0)	7 (0.0)	0 (0.0)	1 (0.0)	0 (0.0)	c diff
heaving	10 (0.0)	6 (0.0)	0 (0.0)	1 (0.0)	0 (0.0)	heaves
effusions	10 (0.0)	6 (0.0)	0 (0.0)	0 (0.0)	2 (0.0)	
diverticulitis	10 (0.0)	1 (0.0)	0 (0.0)	1 (0.0)	1 (0.0)	
bacteria	10 (0.0)	9 (0.0)	0 (0.0)	3 (0.0)	0 (0.0)	
ammonia	10 (0.0)	2 (0.0)	0 (0.0)	0 (0.0)	0 (0.0)	
pericardial effusion	9 (0.0)	4 (0.0)	0 (0.0)	1 (0.0)	2 (0.0)	
splenomegaly	9 (0.0)	4 (0.0)	0 (0.0)	1 (0.0)	4 (0.1)	
stranding	9 (0.0)	5 (0.0)	0 (0.0)	1 (0.0)	0 (0.0)	
troponins	9 (0.0)	4 (0.0)	0 (0.0)	1 (0.0)	2 (0.0)	trop
hypocalcemia	9 (0.0)	8 (0.0)	0 (0.0)	3 (0.0)	1 (0.0)	
nt probnp	8 (0.0)	4 (0.0)	0 (0.0)	0 (0.0)	0 (0.0)	bnp | probnp
cholecystitis	8 (0.0)	3 (0.0)	0 (0.0)	0 (0.0)	0 (0.0)	
inflammatory	8 (0.0)	3 (0.0)	0 (0.0)	0 (0.0)	1 (0.0)	
pneumothorax	8 (0.0)	5 (0.0)	0 (0.0)	3 (0.0)	0 (0.0)	
anion gap	7 (0.0)	3 (0.0)	0 (0.0)	0 (0.0)	1 (0.0)	ag | anion gap metabolic | gap metabolic acidosis
erythrocyte sedimentation rate	7 (0.0)	3 (0.0)	0 (0.0)	2 (0.0)	0 (0.0)	elevated esr | esr

© 2025 Pak TR et al. *JAMA Network Open*

eTable 2 (cont.)

	Patients with labeled admission notes, No. (%)					
	All	And with ≥1 positive culture[a]	And with MRSA in ≥1 culture[a]	And with MDRGN in ≥1 culture[a]	And with in-hospital mortality	All synonyms[b]
pseudomonas	7 (0.0)	3 (0.0)	0 (0.0)	1 (0.0)	0 (0.0)	
uremia	6 (0.0)	4 (0.0)	0 (0.0)	3 (0.0)	0 (0.0)	elevated bun
septic arthritis	6 (0.0)	1 (0.0)	0 (0.0)	0 (0.0)	0 (0.0)	
inflammatory markers	6 (0.0)	5 (0.0)	0 (0.0)	1 (0.0)	0 (0.0)	
klebsiella	6 (0.0)	4 (0.0)	0 (0.0)	1 (0.0)	0 (0.0)	
subdural hemorrhage	6 (0.0)	3 (0.0)	0 (0.0)	0 (0.0)	2 (0.0)	sdh
peritonitis	6 (0.0)	1 (0.0)	0 (0.0)	0 (0.0)	1 (0.0)	
creatine kinase	5 (0.0)	3 (0.0)	0 (0.0)	0 (0.0)	0 (0.0)	ck | elevated ck
blood urea nitrogen	5 (0.0)	4 (0.0)	0 (0.0)	1 (0.0)	1 (0.0)	bun
blood pressure	5 (0.0)	3 (0.0)	0 (0.0)	0 (0.0)	0 (0.0)	sbp | sbps
kidney disease	4 (0.0)	3 (0.0)	0 (0.0)	1 (0.0)	0 (0.0)	renal insufficiency
lipase	4 (0.0)	2 (0.0)	0 (0.0)	0 (0.0)	0 (0.0)	elevated lipase
gram negative rod	4 (0.0)	3 (0.0)	1 (0.1)	0 (0.0)	0 (0.0)	gnr | gnr bacteremia
ekg changes	4 (0.0)	2 (0.0)	0 (0.0)	2 (0.0)	0 (0.0)	
constriction	4 (0.0)	1 (0.0)	0 (0.0)	0 (0.0)	0 (0.0)	
stenosis	4 (0.0)	3 (0.0)	0 (0.0)	1 (0.0)	0 (0.0)	
endocarditis	4 (0.0)	1 (0.0)	0 (0.0)	0 (0.0)	0 (0.0)	
strep	4 (0.0)	3 (0.0)	0 (0.0)	0 (0.0)	0 (0.0)	
staphylococcus	4 (0.0)	1 (0.0)	0 (0.0)	0 (0.0)	0 (0.0)	staph
urine culture	3 (0.0)	1 (0.0)	0 (0.0)	1 (0.0)	0 (0.0)	
inappropriate behavior	3 (0.0)	1 (0.0)	0 (0.0)	0 (0.0)	0 (0.0)	
obesity	3 (0.0)	0 (0.0)	0 (0.0)	0 (0.0)	0 (0.0)	morbid obesity
ct chest	3 (0.0)	2 (0.0)	0 (0.0)	0 (0.0)	0 (0.0)	chest ct | ggos
hepatitis	3 (0.0)	2 (0.0)	0 (0.0)	0 (0.0)	0 (0.0)	
varices	3 (0.0)	2 (0.0)	0 (0.0)	0 (0.0)	1 (0.0)	
post traumatic stress disorder	3 (0.0)	1 (0.0)	0 (0.0)	0 (0.0)	0 (0.0)	ptsd
bipolar disorder	2 (0.0)	0 (0.0)	0 (0.0)	0 (0.0)	0 (0.0)	bipolar
culture	2 (0.0)	2 (0.0)	0 (0.0)	1 (0.0)	0 (0.0)	cultures | cultures positive
hypothyroidism	2 (0.0)	1 (0.0)	0 (0.0)	1 (0.0)	0 (0.0)	
cirrhosis	2 (0.0)	0 (0.0)	0 (0.0)	0 (0.0)	1 (0.0)	
ca	2 (0.0)	2 (0.0)	1 (0.1)	0 (0.0)	0 (0.0)	
mm	2 (0.0)	2 (0.0)	0 (0.0)	0 (0.0)	0 (0.0)	

© 2025 Pak TR et al. *JAMA Network Open*

eTable 2 (cont.)

	Patients with labeled admission notes, No. (%)					
	All	And with ≥1 positive culture[a]	And with MRSA in ≥1 culture[a]	And with MDRGN in ≥1 culture[a]	And with in-hospital mortality	All synonyms[b]
neutrophil count	2 (0.0)	1 (0.0)	0 (0.0)	0 (0.0)	0 (0.0)	anc | neutrophils
nephrolithiasis	2 (0.0)	2 (0.0)	0 (0.0)	0 (0.0)	0 (0.0)	
subarachnoid hemorrhage	2 (0.0)	1 (0.0)	0 (0.0)	1 (0.0)	0 (0.0)	sah
chronic kidney disease	1 (0.0)	1 (0.0)	0 (0.0)	1 (0.0)	0 (0.0)	ckd | esrd
ct abdomen	1 (0.0)	0 (0.0)	0 (0.0)	0 (0.0)	0 (0.0)	ctap
benign prostatic hyperplasia	1 (0.0)	0 (0.0)	0 (0.0)	0 (0.0)	0 (0.0)	bph
human immunodeficiency virus	1 (0.0)	0 (0.0)	0 (0.0)	0 (0.0)	0 (0.0)	hiv
cardiomyopathy	1 (0.0)	0 (0.0)	0 (0.0)	0 (0.0)	0 (0.0)	cm
platelet count	1 (0.0)	0 (0.0)	0 (0.0)	0 (0.0)	0 (0.0)	plt
osteoporosis	1 (0.0)	1 (0.0)	1 (0.1)	0 (0.0)	0 (0.0)	
escherichia coli	0 (0.0)	0 (0.0)	0 (0.0)	0 (0.0)	0 (0.0)	e coli
hepatitis c	0 (0.0)	0 (0.0)	0 (0.0)	0 (0.0)	0 (0.0)	hcv
lactate dehydrogenase	0 (0.0)	0 (0.0)	0 (0.0)	0 (0.0)	0 (0.0)	elevated ldh | ldh
prostate cancer	0 (0.0)	0 (0.0)	0 (0.0)	0 (0.0)	0 (0.0)	
creatinine	0 (0.0)	0 (0.0)	0 (0.0)	0 (0.0)	0 (0.0)	serum creatinine
hyperlipidemia	0 (0.0)	0 (0.0)	0 (0.0)	0 (0.0)	0 (0.0)	hld
obstructive sleep apnea	0 (0.0)	0 (0.0)	0 (0.0)	0 (0.0)	0 (0.0)	osa
mssa	0 (0.0)	0 (0.0)	0 (0.0)	0 (0.0)	0 (0.0)	
hepatomegaly	0 (0.0)	0 (0.0)	0 (0.0)	0 (0.0)	0 (0.0)	
venous blood gas	0 (0.0)	0 (0.0)	0 (0.0)	0 (0.0)	0 (0.0)	vbg
lymphoma	0 (0.0)	0 (0.0)	0 (0.0)	0 (0.0)	0 (0.0)	
schizophrenia	0 (0.0)	0 (0.0)	0 (0.0)	0 (0.0)	0 (0.0)	
complete blood count	0 (0.0)	0 (0.0)	0 (0.0)	0 (0.0)	0 (0.0)	cbc
basic metabolic panel	0 (0.0)	0 (0.0)	0 (0.0)	0 (0.0)	0 (0.0)	bmp
meningitis	0 (0.0)	0 (0.0)	0 (0.0)	0 (0.0)	0 (0.0)	
infarct	0 (0.0)	0 (0.0)	0 (0.0)	0 (0.0)	0 (0.0)	
bicarbonate	0 (0.0)	0 (0.0)	0 (0.0)	0 (0.0)	0 (0.0)	bicarb

© 2025 Pak TR et al. *JAMA Network Open*

eTable 2 (cont.)

Abbreviations: MDRGN, multidrug-resistant gram negative organism; MRSA, methicillin-resistant Staphylococcus aureus.

[a] Cultures collected within 72h of arrival to the emergency department, from any of the following body sites: blood, urine, intra-abdominal fluid, pleural fluid, bronchial or bronchoalveolar lavage fluid, cerebrospinal fluid, pericardial fluid, retropharyngeal fluid, sputum, abscesses, deep tissue, joint spaces, renal stones, or catheter tips.
[b] Synonyms can appear in multiple rows, indicating that multiple sign/symptom labels would be assigned upon matching the synonym, e.g., "febrile neutropenia" assigns both the "fever" and "neutropenia" labels.

© 2025 Pak TR et al. *JAMA Network Open*

eTable 3. Comparative validation of LLM vs ICD-10-CM strategies for extracting the top 30 presenting signs/symptoms against gold standard human chart review.

For details of the ICD-10-CM strategy, see Methods and eTable 1. LLM- and human-generated labels were filtered to the 30 most prevalent signs/symptoms before this comparison, as the ICD-10-CM method was limited to assigning only these 30 labels. As a result, metrics in the first column differ from Table 1.

	**Comparison** Value (95% CI), %	
**Metric**	**LLM method vs primary physician reviewer**	**ICD-10-CM codes vs primary physician reviewer**
Method being validated	LLaMA 3 8B, top 30 signs/symptoms only	ICD-10-CM codes for top 30 signs/symptoms
Gold standard or ground truth method	Primary physician	Primary physician
No. of admission notes compared	303	303
Total No. of possible labels	122,412	122,412
Accuracy	99.7 (99.6–99.7)	99.2 (99.2–99.3)
Balanced accuracy	92.5 (91.3–93.6)	68.6 (66.7–70.3)
Sensitivity	85.2 (82.7–87.3)	37.5 (34.0–41.2)
Specificity	99.8 (99.8–99.8)	99.7 (99.7–99.8)
F1 score	80.8 (78.9–82.8)	44.4 (40.9–48.0)
Positive predictive value	76.9 (74.2–79.4)	54.4 (49.9–58.8)
Negative predictive value	99.9 (99.9–99.9)	99.5 (99.4–99.5)
Cohen κ, No.	0.81 (0.79–0.83)	0.44 (0.41–0.47)

Abbreviations: ICD-10-CM, International Classification of Diseases, Tenth Revision, Clinical Modification; LLM, large language model.

eTable 4. Comparative validation of the original LLM strategy vs an alternative LLM strategy of prompting for five signs/symptoms at a time against gold standard human chart review.

For details of the alternative LLM strategy, see eMethods. Validation results for the original LLM method (from Table 1 in the manuscript) are presented in the first column for comparison.

	**Comparison**<br>Value (95% CI), %	
**Metric**	**Original method vs primary physician reviewer**	**Five signs/symptoms at a time vs primary physician reviewer**
Method being validated	LLaMA 3 8B, original method	LLaMA 3 8B, up to five signs/symptoms at a time
Gold standard or ground truth method	Primary physician	Primary physician
No. of admission notes compared	303	303
Total No. of possible labels	122,412	122,412
Accuracy	99.3 (99.2–99.3)	89.0 (88.8–89.2)
Balanced accuracy	84.6 (83.5–85.8)	91.5 (90.8–92.1)
Sensitivity	69.7 (67.3–72.0)	94.1 (92.7–95.2)
Specificity	99.6 (99.6–99.6)	88.9 (88.7–89.1)
F1 score	69.0 (67.1–70.9)	17.0 (16.2–17.8)
Positive predictive value	68.4 (66.0–70.7)	9.3 (8.9–9.8)
Negative predictive value	99.6 (99.6–99.7)	99.9 (99.9–99.9)
Cohen κ, No.	0.69 (0.67–0.70)	0.15 (0.14–0.16)

Abbreviations: LLM, large language model.

© 2025 Pak TR et al. *JAMA Network Open*

eTable 5. Thirty most prevalent signs/symptoms in possible infection admissions and proportions for each sign/symptom of culture positivity and mortality.

	Patients with labeled admission notes, No. (%)				
	**All**	**≥1 positive culture[a]**	**MRSA in ≥1 culture[a]**	**MDRGN in ≥1 culture[a]**	**Ending in mortality**
**By sign/symptom**					
fever	36,286 (100)	21,666 (59.7)	815 (2.2)	3,847 (10.6)	1,025 (2.8)
dyspnea	25,572 (100)	14,864 (58.1)	579 (2.3)	1,966 (7.7)	2,069 (8.1)
cough	24,018 (100)	14,600 (60.8)	534 (2.2)	1,659 (6.9)	1,225 (5.1)
abdominal pain	20,831 (100)	11,209 (53.8)	236 (1.1)	2,088 (10.0)	784 (3.8)
pain	20,300 (100)	9,491 (46.8)	492 (2.4)	1,447 (7.1)	479 (2.4)
nausea	20,036 (100)	10,881 (54.3)	252 (1.3)	1,773 (8.8)	648 (3.2)
chills	19,923 (100)	11,725 (58.9)	362 (1.8)	2,094 (10.5)	343 (1.7)
fatigue	19,439 (100)	11,424 (58.8)	268 (1.4)	1,789 (9.2)	1,020 (5.2)
vomiting	17,000 (100)	9,307 (54.7)	227 (1.3)	1,595 (9.4)	641 (3.8)
altered mental status	16,010 (100)	10,033 (62.7)	341 (2.1)	2,024 (12.6)	1,304 (8.1)
diarrhea	11,941 (100)	6,329 (53.0)	161 (1.3)	971 (8.1)	506 (4.2)
chest pain	9,503 (100)	5,230 (55.0)	221 (2.3)	632 (6.7)	459 (4.8)
weakness	9,212 (100)	5,416 (58.8)	143 (1.6)	912 (9.9)	489 (5.3)
swelling	8,817 (100)	3,351 (38.0)	321 (3.6)	343 (3.9)	188 (2.1)
sputum changes	8,080 (100)	5,692 (70.4)	269 (3.3)	752 (9.3)	378 (4.7)
redness	7,928 (100)	2,645 (33.4)	301 (3.8)	289 (3.6)	86 (1.1)
headache	7,335 (100)	3,932 (53.6)	111 (1.5)	489 (6.7)	148 (2.0)
poor appetite	7,275 (100)	4,274 (58.7)	105 (1.4)	670 (9.2)	367 (5.0)
malaise	7,207 (100)	4,308 (59.8)	126 (1.7)	665 (9.2)	263 (3.6)
back pain	5,974 (100)	3,582 (60.0)	151 (2.5)	530 (8.9)	215 (3.6)
hypoxemia	4,921 (100)	3,111 (63.2)	169 (3.4)	552 (11.2)	716 (14.5)
hypotension	4,093 (100)	2,505 (61.2)	109 (2.7)	543 (13.3)	527 (12.9)
dizziness	4,009 (100)	2,110 (52.6)	39 (1.0)	280 (7.0)	141 (3.5)
dysuria	3,964 (100)	3,087 (77.9)	24 (0.6)	629 (15.9)	59 (1.5)
rhinorrhea	3,931 (100)	2,308 (58.7)	57 (1.5)	246 (6.3)	123 (3.1)
lightheadedness	3,546 (100)	1,868 (52.7)	36 (1.0)	241 (6.8)	146 (4.1)
fall	3,338 (100)	1,931 (57.8)	44 (1.3)	304 (9.1)	206 (6.2)
urinary frequency	3,321 (100)	2,494 (75.1)	28 (0.8)	486 (14.6)	55 (1.7)
diaphoresis	3,225 (100)	1,779 (55.2)	72 (2.2)	299 (9.3)	92 (2.9)
leg swelling	3,100 (100)	1,296 (41.8)	50 (1.6)	155 (5.0)	160 (5.2)

Abbreviations: MDRGN, multidrug-resistant gram negative organism; MRSA, methicillin-resistant *Staphylococcus aureus*.

[a] Cultures collected within 72h of arrival to the emergency department, from any of the following body sites: blood, urine, intra-abdominal fluid, pleural fluid, bronchial or bronchoalveolar lavage fluid, cerebrospinal fluid, pericardial fluid, retropharyngeal fluid, sputum, abscesses, deep tissue, joint spaces, renal stones, or catheter tips.

© 2025 Pak TR et al. *JAMA Network Open*

eTable 6. Crude relative risk of MRSA culture positivity for each of the top 30 signs/symptoms.

Sign/symptom	Count				RR	RR 95% CI[a]		Adjusted P-value[a]
	With the sign/symptom		Without the sign/symptom					
	With MRSA	Without MRSA	With MRSA	Without MRSA		Low	High	
redness	301	2344	1602	47780	3.51	2.91	4.23	<0.001
swelling	321	3030	1582	47094	2.95	2.45	3.54	<0.001
pain	492	8999	1411	41125	1.56	1.33	1.84	<0.001
hypoxemia	169	2942	1734	47182	1.53	1.20	1.96	<0.001
sputum changes	269	5423	1634	44701	1.34	1.09	1.64	<0.001
hypotension	109	2396	1794	47728	1.20	0.89	1.63	1.000
chest pain	221	5009	1682	45115	1.18	0.94	1.47	0.632
back pain	151	3431	1752	46693	1.17	0.90	1.51	1.000
diaphoresis	72	1707	1831	48417	1.11	0.77	1.61	1.000
dyspnea	579	14285	1324	35839	1.09	0.94	1.27	1.000
leg swelling	50	1246	1853	48878	1.06	0.68	1.64	1.000
fever	815	20851	1088	29273	1.05	0.91	1.21	1.000
cough	534	14066	1369	36058	1.00	0.85	1.17	1.000
altered mental status	341	9692	1562	40432	0.91	0.76	1.10	1.000
chills	362	11363	1541	38761	0.81	0.67	0.97	0.006
malaise	126	4182	1777	45942	0.79	0.59	1.04	0.224
headache	111	3821	1792	46303	0.76	0.56	1.03	0.112
weakness	143	5273	1760	44851	0.70	0.53	0.92	<0.001
diarrhea	161	6168	1742	43956	0.67	0.52	0.86	<0.001
rhinorrhea	57	2251	1846	47873	0.67	0.44	1.01	0.056
poor appetite	105	4169	1798	45955	0.65	0.48	0.89	<0.001
vomiting	227	9080	1676	41044	0.62	0.50	0.77	<0.001
fall	44	1887	1859	48237	0.61	0.38	0.99	0.030
fatigue	268	11156	1635	38968	0.58	0.47	0.71	<0.001
nausea	252	10629	1651	39495	0.58	0.47	0.71	<0.001
lightheadedness	36	1832	1867	48292	0.52	0.31	0.87	0.001
abdominal pain	236	10973	1667	39151	0.52	0.42	0.64	<0.001
dizziness	39	2071	1864	48053	0.49	0.30	0.82	<0.001
urinary frequency	28	2466	1875	47658	0.30	0.16	0.54	<0.001
dysuria	24	3063	1879	47061	0.20	0.11	0.39	<0.001

Abbreviations: MRSA, methicillin-resistant *Staphylococcus aureus*; RR, relative risk.

[a] After Bonferroni adjustment for 30 signs/symptoms tested.

© 2025 Pak TR et al. *JAMA Network Open*

eTable 7. Crude relative risk of MDR gram-negative culture positivity for each of the top 30 signs/symptoms.

Sign/symptom	Count				RR	RR 95% CI[a]		Adjusted P-value[a]
	With the sign/symptom		Without the sign/symptom					
	With MDRGN	Without MDRGN	With MDRGN	Without MDRGN		Low	High	
hypotension	543	1962	8074	41448	1.33	1.17	1.50	<0.001
altered mental status	2024	8009	6593	35401	1.28	1.20	1.38	<0.001
dysuria	629	2458	7988	40952	1.25	1.11	1.40	<0.001
urinary frequency	486	2008	8131	41402	1.19	1.04	1.35	0.002
abdominal pain	2088	9121	6529	34289	1.16	1.08	1.25	<0.001
fever	3847	17819	4770	25591	1.13	1.06	1.20	<0.001
chills	2094	9631	6523	33779	1.10	1.03	1.19	<0.001
hypoxemia	552	2559	8065	40851	1.08	0.95	1.22	1.000
vomiting	1595	7712	7022	35698	1.04	0.96	1.13	1.000
weakness	912	4504	7705	38906	1.02	0.92	1.13	1.000
diaphoresis	299	1480	8318	41930	1.02	0.86	1.20	1.000
nausea	1773	9108	6844	34302	0.98	0.91	1.06	1.000
fall	304	1627	8313	41783	0.95	0.80	1.12	1.000
poor appetite	670	3604	7947	39806	0.94	0.84	1.06	1.000
fatigue	1789	9635	6828	33775	0.93	0.86	1.01	0.099
malaise	665	3643	7952	39767	0.93	0.82	1.04	1.000
diarrhea	971	5358	7646	38052	0.92	0.83	1.01	0.160
pain	1447	8044	7170	35366	0.90	0.83	0.98	0.004
back pain	530	3052	8087	40358	0.89	0.78	1.01	0.096
dizziness	280	1830	8337	41580	0.79	0.67	0.95	<0.001
sputum changes	752	4940	7865	38470	0.78	0.70	0.87	<0.001
lightheadedness	241	1627	8376	41783	0.77	0.64	0.94	<0.001
dyspnea	1966	12898	6651	30512	0.74	0.69	0.80	<0.001
headache	489	3443	8128	39967	0.74	0.64	0.84	<0.001
leg swelling	155	1141	8462	42269	0.72	0.56	0.91	<0.001
chest pain	632	4598	7985	38812	0.71	0.63	0.80	<0.001
redness	289	2356	8328	41054	0.65	0.54	0.77	<0.001
rhinorrhea	246	2062	8371	41348	0.63	0.52	0.77	<0.001
cough	1659	12941	6958	30469	0.61	0.56	0.66	<0.001
swelling	343	3008	8274	40402	0.60	0.51	0.71	<0.001

Abbreviations: MDR, multidrug-resistant; MDRGN, multidrug-resistant gram-negative organisms; RR, relative risk.

[a] After Bonferroni adjustment for 30 signs/symptoms tested.

© 2025 Pak TR et al. *JAMA Network Open*

eTable 8. Crude relative risk of in-hospital mortality for each of the top 30 signs/symptoms.

Sign/symptom	Count: With the sign/symptom: Deceased	Count: With the sign/symptom: Alive	Count: Without the sign/symptom: Deceased	Count: Without the sign/symptom: Alive	RR	RR 95% CI[a]: Low	RR 95% CI[a]: High	Adjusted P-value[a]
hypoxemia	716	3417	3748	64888	3.17	2.82	3.57	<0.001
hypotension	527	3018	3937	65287	2.61	2.28	2.99	<0.001
dyspnea	2069	18297	2395	50008	2.22	2.03	2.43	<0.001
altered mental status	1304	11521	3160	56784	1.93	1.75	2.13	<0.001
fall	206	2261	4258	66044	1.38	1.11	1.71	<0.001
weakness	489	6658	3975	61647	1.13	0.98	1.31	0.260
fatigue	1020	14130	3444	54175	1.13	1.01	1.26	0.017
leg swelling	160	2165	4304	66140	1.13	0.88	1.44	1.000
poor appetite	367	5160	4097	63145	1.09	0.92	1.29	1.000
chest pain	459	6646	4005	61659	1.06	0.91	1.23	1.000
cough	1225	18270	3239	50035	1.03	0.93	1.14	1.000
sputum changes	378	6252	4086	62053	0.92	0.78	1.09	1.000
diarrhea	506	8413	3958	59892	0.92	0.79	1.06	1.000
lightheadedness	146	2463	4318	65842	0.91	0.70	1.18	1.000
abdominal pain	784	14383	3680	53922	0.81	0.72	0.91	<0.001
back pain	215	4093	4249	64212	0.80	0.65	1.00	0.038
vomiting	641	12086	3823	56219	0.79	0.69	0.90	<0.001
dizziness	141	2862	4323	65443	0.76	0.58	0.99	0.024
malaise	263	5531	4201	62774	0.72	0.60	0.88	<0.001
nausea	648	14259	3816	54046	0.66	0.58	0.75	<0.001
rhinorrhea	123	3044	4341	65261	0.62	0.47	0.83	<0.001
diaphoresis	92	2407	4372	65898	0.59	0.43	0.82	<0.001
pain	479	14578	3985	53727	0.46	0.40	0.53	<0.001
fever	1025	28990	3439	39315	0.42	0.38	0.47	<0.001
headache	148	5314	4316	62991	0.42	0.33	0.55	<0.001
swelling	188	6836	4276	61469	0.41	0.33	0.52	<0.001
urinary frequency	55	2648	4409	65657	0.32	0.21	0.49	<0.001
chills	343	15875	4121	52430	0.29	0.24	0.35	<0.001
dysuria	59	3183	4405	65122	0.29	0.19	0.43	<0.001
redness	86	6589	4378	61716	0.19	0.14	0.27	<0.001

Abbreviations: RR, relative risk.

[a] After Bonferroni adjustment for 30 signs/symptoms tested.

© 2025 Pak TR et al. *JAMA Network Open*

eTable 9. Adjusted odds ratios for culture positivity for MRSA, culture positivity for MDRGN organisms, and in-hospital mortality for each syndrome.

Outcome variable	Syndrome	N	Adjusted OR	aOR 95% CI[a]		Adjusted P-value[a]
				Low	High	
MRSA positivity	skin and soft tissue	13015	1.727	1.490	2.001	<0.001
	gastrointestinal	21485	0.629	0.541	0.732	<0.001
	back pain	3582	1.166	0.914	1.487	0.627
	urinary tract	4766	0.336	0.225	0.502	<0.001
	dizziness	3500	0.676	0.480	0.953	0.015
	cardiopulmonary	31723	0.863	0.739	1.008	0.075
	constitutional	35429	0.907	0.785	1.047	0.474
MDRGN positivity	skin and soft tissue	13015	0.853	0.786	0.926	<0.001
	gastrointestinal	21485	1.136	1.058	1.219	<0.001
	back pain	3582	0.928	0.811	1.063	0.978
	urinary tract	4766	1.261	1.130	1.407	<0.001
	dizziness	3500	0.742	0.644	0.856	<0.001
	cardiopulmonary	31723	0.761	0.706	0.820	<0.001
	constitutional	35429	1.124	1.042	1.213	<0.001
In-hospital mortality	skin and soft tissue	28319	1.015	0.910	1.132	1
	gastrointestinal	38603	0.923	0.840	1.015	0.164
	back pain	5974	1.136	0.935	1.379	0.548
	urinary tract	6285	0.714	0.552	0.923	0.003
	dizziness	6656	0.929	0.782	1.104	1
	cardiopulmonary	53478	1.301	1.168	1.449	<0.001
	constitutional	61004	0.834	0.761	0.913	<0.001

Abbreviations: aOR, adjusted odds ratio; MRSA, methicillin-resistant *Staphylococcus aureus*; MDRGN, multidrug-resistant gram-negative organisms; OR, odds ratio.

[a] After Bonferroni adjustment for 7 syndromes tested

eReferences

1. Tamma PD, Heil EL, Justo JA, Mathers AJ, Satlin MJ, Bonomo RA. Infectious Diseases Society of America 2024 guidance on the treatment of antimicrobial-resistant gram-negative infections. *Clin Infect Dis*. Published online August 7, 2024:ciae403.

2. Van Walraven C, Austin PC, Jennings A, Quan H, Forster AJ. A modification of the elixhauser comorbidity measures into a point system for hospital death using administrative data. *Med Care*. 2009;47(6):626-633.

3. Pak TR, Young J, McKenna CS, et al. Risk of Misleading Conclusions in Observational Studies of Time-to-Antibiotics and Mortality in Suspected Sepsis. *Clin Infect Dis*. 2023;77(11):1534-1543.

4. Pak TR, Sánchez SM, McKenna CS, Rhee C, Klompas M. Assessment of racial, ethnic, and sex-based disparities in time-to-antibiotics and sepsis outcomes in a large multihospital cohort. *Crit Care Med*. 2024;52(12):1928-1933.

5. Murtagh F, Legendre P. Ward's hierarchical agglomerative clustering method: Which algorithms implement ward's criterion? *J Classif*. 2014;31(3):274-295.

© 2025 Pak TR et al. *JAMA Network Open*
