# Supplementary material for: Syndromic Analysis of Sepsis Cohorts Using Large Language Models
Source: JAMA Netw Open. 2025 Oct 24;8(10):e2539267. doi: 10.1001/jamanetworkopen.2025.39267 (PMC12552932; doi:10.1001/jamanetworkopen.2025.39267)
Supplement: Supplement 2. — Data Sharing Statement [file jamanetwopen-e2539267-s002.pdf]

Data Sharing Statement

Pak. Syndromic Analysis of Sepsis Cohorts Using Large Language Models. *JAMA Netw Open*. Published October 24, 2025. doi:10.1001/jamanetworkopen.2025.39267

Data
Data available: No

Additional Information
Explanation for why data not available: The individual patient data for this study includes over 90,000 full-length clinical notes, which cannot be readily de-identified for sharing outside of our health system. However, the Python package we developed to implement the LLM clinical information extraction procedure will be made available on Github at https://github.com/tpaklab/llacie at the time of publication.
